# A new Bayesian factor analysis method improves detection of genes and biological processes affected by perturbations in single-cell CRISPR screening

**DOI:** 10.1038/s41592-023-02017-4

**Published:** 2023-09-28

**Authors:** Yifan Zhou, Kaixuan Luo, Lifan Liang, Mengjie Chen, Xin He

**Affiliations:** 1 Graduate Program of Biophysical Sciences, University of Chicago, Chicago, IL USA; 2 Department of Human Genetics, University of Chicago, Chicago, IL USA; 3 Department of Medicine, University of Chicago, Chicago, IL USA

**Keywords:** Statistical methods, Gene regulation, High-throughput screening, Transcriptomics, Gene expression

## Abstract

Clustered regularly interspaced short palindromic repeats (CRISPR) screening coupled with single-cell RNA sequencing has emerged as a powerful tool to characterize the effects of genetic perturbations on the whole transcriptome at a single-cell level. However, due to its sparsity and complex structure, analysis of single-cell CRISPR screening data is challenging. In particular, standard differential expression analysis methods are often underpowered to detect genes affected by CRISPR perturbations. We developed a statistical method for such data, called guided sparse factor analysis (GSFA). GSFA infers latent factors that represent coregulated genes or gene modules; by borrowing information from these factors, it infers the effects of genetic perturbations on individual genes. We demonstrated through extensive simulation studies that GSFA detects perturbation effects with much higher power than state-of-the-art methods. Using single-cell CRISPR data from human CD8^+^ T cells and neural progenitor cells, we showed that GSFA identified biologically relevant gene modules and specific genes affected by CRISPR perturbations, many of which were missed by existing methods, providing new insights into the functions of genes involved in T cell activation and neurodevelopment.

## Abstract (web summary)

Guided sparse factor analysis (GSFA) is a powerful statistical framework to detect changes in gene expression as a result of perturbations in single-cell CRISPR screening.

## Main

The discovery of CRISPR and development of the CRISPR–Cas9 system for genomic editing has revolutionized biology^1,2^. A powerful application of the CRISPR–Cas9 system is pooled CRISPR screening, where many genes or genomic sites are edited at the same time to screen for genes with certain functions. This approach has enabled the discovery of many genes involved in processes such as cell proliferation and survival, immune responses and drug resistance^3–5^. Technologies such as CROP sequencing (CROP-seq)^6^ and Perturb sequencing (Perturb-seq)^7^ combine the multiplexed CRISPR screening approach with single-cell RNA sequencing (scRNA-seq), providing comprehensive molecular readouts of the target perturbations within single cells. Single-cell CRISPR screening technologies have found many applications in studies of cellular differentiation, immune responses and regulatory elements^8–11^.

Nevertheless, the analysis of single-cell CRISPR screening data is challenging. Standard differential gene expression (DGE) analysis^12–14^, when applied to single-cell screening data, can be underpowered because of the sparsity and noise inherent to scRNA-seq data, and the relatively small numbers of cells per perturbation (often hundreds or less) in typical experiments. Another commonly used analysis method is clustering cells based on their transcriptome similarity and then assessing whether cells with a specific perturbation are enriched or depleted in any cluster^10,15^. However, the clustering approach has a conceptual flaw. Cell clustering patterns may be driven by multiple biological processes. Even if a perturbation is associated with a cluster, it does not necessarily mean that the perturbation affects all the genes or biological processes associated with that cluster, a point we demonstrate with simulations. Thus, this clustering-based approach does not explicitly link the perturbations with the affected genes. Given the limitations of standard DGE and clustering-based analyses, statistical methods that accommodate the unique features and complexities of single-cell CRISPR screening data are greatly needed.

Our proposed approach is motivated by the observation that genetic perturbations typically affect expression, not one gene at a time, but many related genes simultaneously. Indeed, single-cell CRISPR experiments often target key regulators such as transcription factors, which coordinate the expression of many genes. These ‘gene modules’ can be inferred by matrix factorization and related techniques^16–23^. We propose inferring gene modules from scRNA-seq data and borrowing information across genes to improve the power of detecting DEGs. Existing factor analysis methods, however, are not readily applied to single-cell CRISPR screening data because the factors are not directly linked with genetic perturbation and the effects of perturbation on individual genes are not assessed.

In this study, we present guided sparse factor analysis (GSFA), a framework for analyzing single-cell CRISPR screening data that bridges factor analysis and differential expression analysis. GSFA assumes the effects of genetic perturbations are mediated through a set of gene modules, mathematically represented as latent factors. GSFA evaluates associations of the genetic perturbations with these latent factors, providing information on the module-level effects of the perturbations. Compared with single-gene differential expression analysis, this factor association analysis may be more sensitive. Indeed, expression of a single gene is influenced by potentially many sources; in contrast, latent factors represent main dimensions of variation of many genes and can be thought of as ‘denoised’ versions of gene expression. While our approach is formulated in terms of latent factors, we still summarize the effects of a perturbation on individual genes as the sum of effects mediated by all the factors. We benchmarked our method through extensive simulation studies and real data applications. GSFA identifies biologically relevant modules and has better power to detect differentially expressed genes (DEGs) than alternative methods, providing insights into the biology of T cell activation and neuronal differentiation.

## Results

### Overview of GSFA

**Fig. 1:**
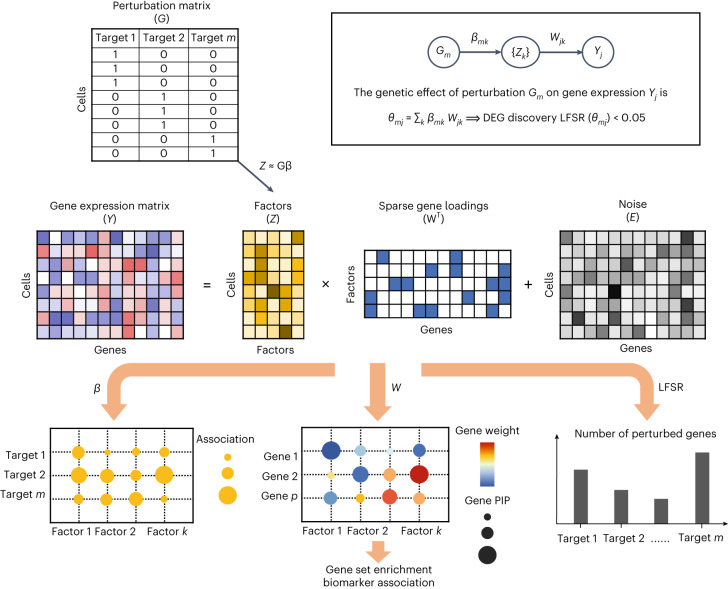
GSFA model and its application on real data. Top, the input of the GSFA includes the perturbation matrix and the gene expression matrix. Bottom, the output of GSFA includes the effects of perturbations on targets (*β*), the gene loading matrix (*W*) and the list of genes affected by each perturbation after LFSR thresholding. The box shows how the GSFA calculates the total effect of a perturbation on the expression of individual genes.

GSFA is a Bayesian statistical model that unifies factor analysis and estimation of the effects of target perturbations. The input of GSFA consists of two matrices: a normalized gene expression matrix across cells; and a ‘perturbation matrix’ that records guide RNA (gRNA) perturbations in each cell (Fig. 1). GSFA assumes that the perturbation of a target gene affects certain latent factors, which in turn changes the expression of individual genes. These assumptions lead to a two-layer model. In the first layer, the expression matrix (*Y*) is decomposed into the product of the factor matrix (*Z*) and the weights of genes on factors (gene loading, *W*). In the second layer, GSFA captures the dependency of factors (*Z*) on perturbations (*G*) via a multivariate linear regression model (Fig. 1).

The main unknowns of the model are the factor matrix (*Z*), the gene loading on factors (*W*) and the effects of perturbations on the factors (*β*). We assume a standard normal prior distribution of *Z* and a ‘spike-and-slab’ prior of *β*, assuming that the effects come from either a normal distribution or a point mass at 0 (ref. ^24^). This sparse prior of *β* encodes the intuition that a genetic perturbation probably affects only a small number of factors. For the gene loading matrix *W*, we also used a sparse prior to limit the number of genes contributing to a factor, facilitating the biological interpretation of factors. We evaluated two choices, the standard spike-and-slab prior and a normal-mixture prior (Methods), where the effect is sampled from a mixture of two normal distributions, one ‘foreground’ component capturing true effects and the other a ‘background’ component absorbing small effects^25,26^. The normal-mixture prior led to better results in our simulations, so it was used as our default prior.

We used a Gibbs sampling algorithm to obtain posterior samples of the model parameters. For any parameter with a sparse prior, the probability that it was sampled from the sparse component was denoted as a posterior inclusion probability (PIP). PIPs quantify whether a perturbation affects a certain factor or whether a gene has loading on a factor. The factors can then be interpreted, for example, through gene ontology (GO) enrichment analysis of genes loaded on the factors. However, when a perturbation affects multiple factors, it can be difficult to synthesize its effects across all affected factors. GSFA provides a way to integrate information over all factors to calculate the total effect of a target perturbation on individual genes. This total effect is the product of the perturbation-to-factor effects and the gene-on-factor loading, summed over all factors (Fig. 1). The significance of the summarized total effect is evaluated using a local false sign rate (LFSR)^27^, a summary of the posterior distribution similar to a local false discovery rate (LFDR) (Methods). The number of factors, *K*, is a user-defined parameter. We provide guidance on the selection of *K* based on how much variance of gene expression is explained by the latent factors (Supplementary Note 4).

In applying GSFA to scRNA-seq data, we first converted the raw unique molecular identifier (UMI) counts into deviance residuals^28^, a continuous quantity analogous to *z*-scores. Compared to the commonly used log transformation, the deviance residual transformation improves the downstream analyses, such as feature selection and clustering (Supplementary Note 2.1). In the CRISPR experiments, negative control gRNAs are often introduced to capture the nonspecific effects of gRNAs. GSFA allows one to remove nonspecific effects by comparing target gRNAs versus negative control gRNAs (Methods). GSFA produces three main outputs (Fig. 1, bottom): the association between genetic perturbations and factors; the weights of genes on factors measured by PIPs; and a list of DEGs of each perturbation at a given LFSR cutoff. In cases where the experiment involves multiple cell types or conditions, GSFA can produce different DEGs for each cell type or condition separately (Supplementary Note 3.2).

### Simulation study demonstrates the advantages of GSFA

We evaluated the performance of GSFA under two settings. In the first simulation setting, referred to as the ‘normal distribution scenario’, we generated continuous gene expression levels with a normal error distribution according to the GSFA model (Methods). Each dataset consisted of 4,000 cells, 6,000 genes, six types of perturbations and ten latent factors. Each perturbation occurs in approximately 5% of cells, mimicking real multiplex CRISPR screening assays. The proportion of genes with nonzero effects on each factor, referred to as factor density, varies from 5% to 20%. For simplicity, each perturbation is associated with a distinct factor. The second ‘count-based’ simulation setting mimics real scRNA-seq UMI data. We converted normally distributed expression levels into count data according to Poisson distributions (Methods). Other simulation parameters remained the same.

**Extended Data Fig. 1:**
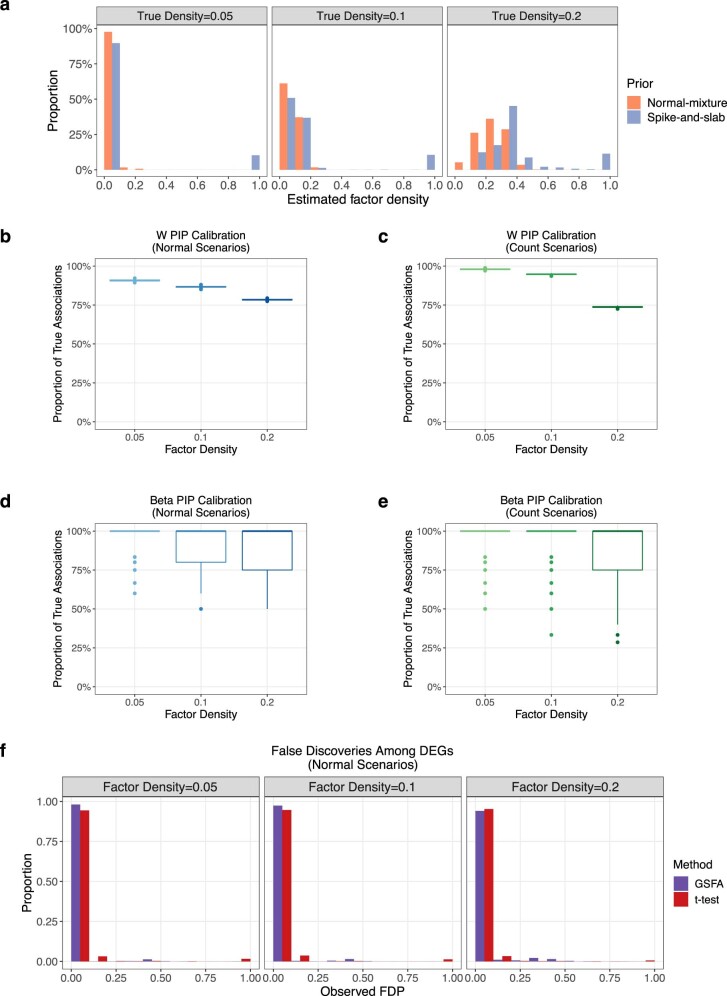
Additional GSFA results on simulated data. **a)** Comparison of estimated factor densities using two priors under the count-based setting. **b)** The proportion of truly associated factor-gene pairs out of all the pairs that have GSFA estimated gene loading PIP > 0.95 in the corresponding factor, computed for each dataset under three levels of true factor density and the normal setting. **c)** Same as in b) but under the count-based setting. **d)** The proportion of truly associated perturbation-factor pairs out of all the pairs that have GSFA estimated association PIP > 0.95, computed for each dataset under three levels of true factor density and the normal setting. **e)** Same as in d) but under the count-based setting. For each box in b), c), d) and e), n = 300 proportion values generated from 300 rounds of simulation under the given setting; the center line of the box represents the median; the lower and upper hinges of the box correspond to the first and third quartiles; the upper/lower whisker extends from the hinge to the largest/smallest value no further than 1.5 * inter-quartile range from the hinge. **f)** Observed proportion of false discoveries among significant DEGs detected by GSFA (LFSR < 0.05) or Welch’s *t*-test (FDR < 0.05), computed for each dataset under three levels of true factor density and the normal setting. Source data

Simulated data allowed us to evaluate model choice, particularly the prior distribution on gene weights (*W*) in count-based data. From our simulations, factors inferred under the spike-and-slab prior sometimes resulted in factors much denser than the ground truth, while the normal-mixture prior led to sparser gene weights (Extended Data Fig. 1a). This justifies our choice of normal-mixture prior as the default prior for read count data.

**Fig. 2:**
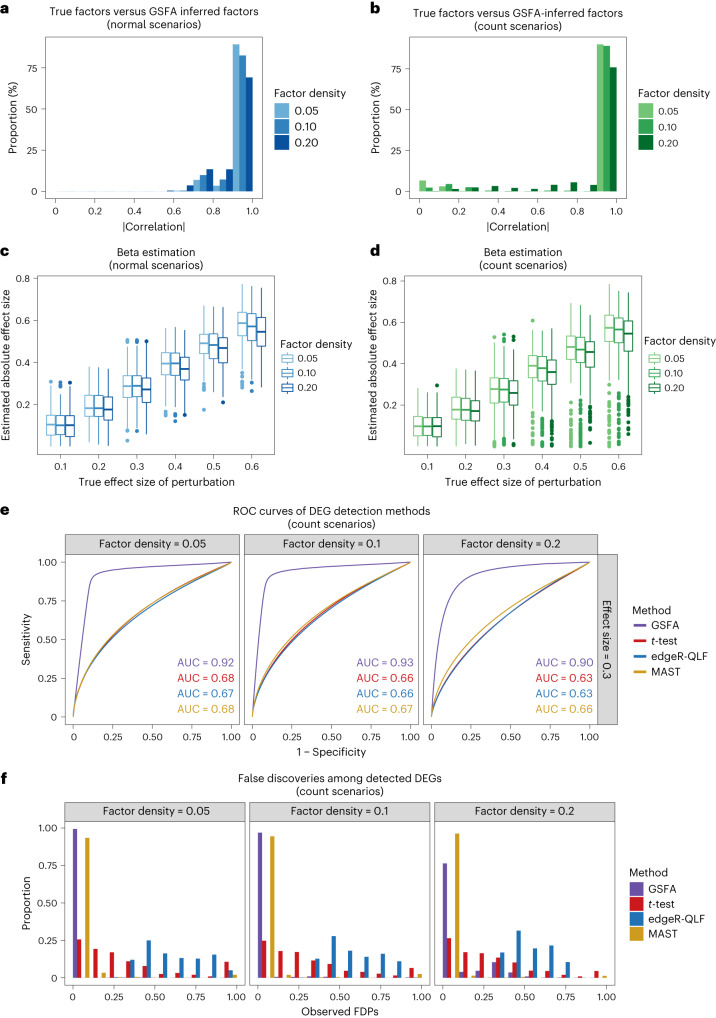
GSFA performance on simulated data. **a**, Distributions of the absolute correlation values between true factors and the factors inferred by GSFA under the normal setting. The different colors represent different values of true factor density varying from 0.05 to 0.2. **b**, Same as in **a** but under count-based scenarios. **c**, Box plots of absolute effect sizes from perturbation factor regression estimated by GSFA under the normal setting. The different colors represent different values of true factor density varying from 0.05 to 0.2. For each box, *n* = 300 estimates generated from 300 rounds of simulation under the given setting; the center line of the box represents the median; the lower and upper hinges of the box correspond to the first and third quartiles; the upper and lower whiskers extend from the hinge to the largest and smallest values no further than 1.5× the interquartile range from the hinge. **d**, Same as in **c** but under count-based scenarios. **e**, Receiver operating characteristic (ROC) curves of DEG discovery under the count-based setting and three different levels of true factor density; the four colors correspond to four DEG detection methods. The results shown are of perturbations with a true association effect of 0.3 on factors. Each curve was a mean representation over 300 datasets generated under the corresponding setting, with the mean area under the curve (AUC) labeled in colored text. See Supplementary Figs. 1 and 2 for results under other settings. **f**, Distributions of the observed FDPs among significant DEGs detected using GSFA (LFSR < 0.05) and other methods (FDR < 0.05) per dataset under the count-based setting and several true factor densities. The four colors correspond to four DEG detection methods. Source data

To evaluate the performance of GSFA in factor inference, we quantified the correlation between inferred and true factors. Across all scenarios, inferred factors were highly correlated with true factors (Fig. 2a,b). GSFA also recovered genes with nonzero loading on the factors. Indeed, genes with PIPs above 0.95 were generally true genes, with observed false discovery proportions (FDPs) below 0.1 when the true factor density was less than 0.2 (Extended Data Fig. 1b,c).

Next, we evaluated the performance of GSFA in detecting the effects of perturbations on factors. Across all scenarios, GSFA estimated these effects accurately (Fig. 2c,d). A small downward bias of estimated effects was expected, given the sparse prior we imposed. We further assessed the calibration of the PIPs of these effects. At a PIP threshold of 0.95 and a true factor density level below 0.2, the proportion of falsely detected effects was generally below 0.1 (Extended Data Fig. 1d,e).

We then compared the performance of GSFA in detecting genes affected by perturbations, with commonly used DEG analysis methods: the Welch’s *t*-test^29^; the edgeR quasi-likelihood *F*-test (edgeR-QLF)^13^; and MAST, a method designed for single-cell analysis^30^. GSFA outperformed the other methods in both sensitivity and specificity under all scenarios (Fig. 2e and Supplementary Figs. 1 and 2). In addition, DEGs detected by GSFA at an LFSR < 0.05 have observed FDPs well below 0.05 in most cases, while edgeR and *t*-test DEGs show substantial inflation under the count-based scenarios (Fig. 2f and Extended Data Fig. 1f).

In the GSFA results presented so far, we used the true value of *K* (ten), the number of factors. We verified that our procedure of selecting *K* led to an estimated value close to ten, and the results were generally robust to *K* (Supplementary Fig. 3).

**Extended Data Fig. 2:**
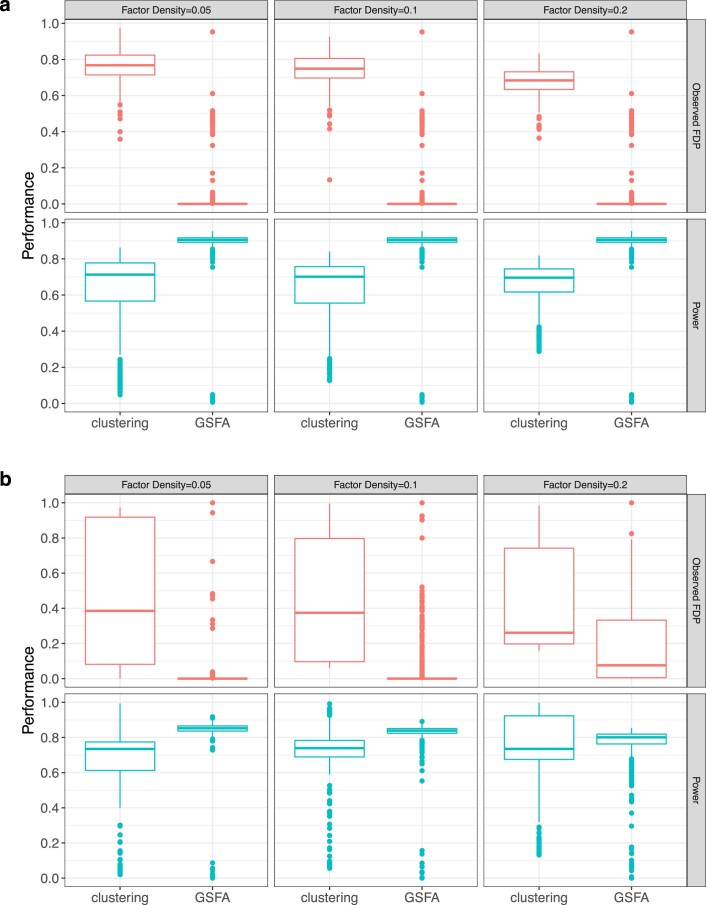
Simulation results of two-step clustering analysis vs. GSFA. Panel **a)** shows Normal based simulation. The first row of Panel a) shows the false positive rates of the discovered DEGs across different factor density settings. The second row shows the power of detecting associations of a guide with a cluster or factor. The clustering method here is based on K-means. For each box, n = 300 estimates generated from 300 rounds of simulation under the given setting; the center line of the box represents the median; the lower and upper hinges of the box correspond to the first and third quartiles; the upper/lower whisker extends from the hinge to the largest/smallest value no further than 1.5 * inter-quartile range from the hinge. Panel **b)** Same as in a) but under the count-based setting. Clustering analysis was done using Seurat. Source data

In addition, we used the simulations to compare GSFA with a commonly used clustering-based procedure, where one clusters cells first and then detects associations of perturbations with clusters. We thought this approach may lead to misleading results. To see this, we defined a list of likely target genes for each perturbation based on clustering. Specifically, for each perturbation, we found all clusters associated with that perturbation, obtained the DEGs of each cluster by comparing the cluster with the others and finally took the union of DEGs from all associated clusters of that perturbation to generate potential target genes. The resulting lists were compared with the true target genes of the perturbations. We found that this two-step clustering approach had high false positive rates, often above 50%, in our simulations (Extended Data Fig. 2). Additionally, the power of the clustering approach is substantially lower than GSFA (Extended Data Fig. 2). These results highlight the weakness of clustering-based analysis and the advantages of GSFA.

**Extended Data Fig. 3:**
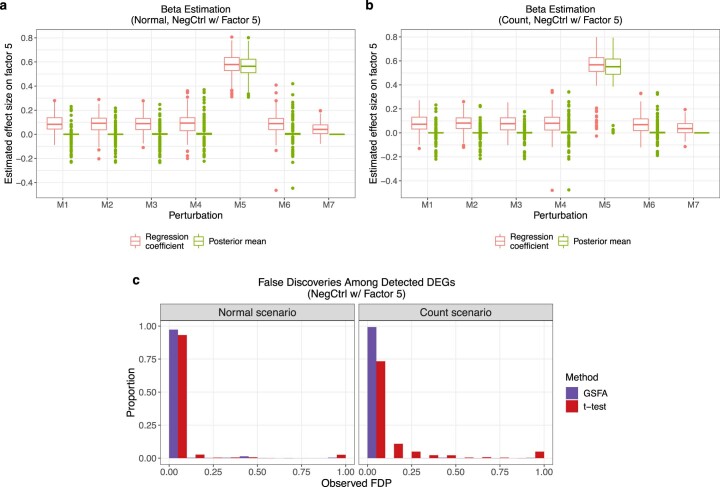
Simulation results under the setting where gRNAs have non-specific effects (see Methods). **a**, **b)** Estimation of beta under the normal scenario (a) and the count-based scenario (b). M1-M6, perturbations; M7, negative control. The true effect sizes, after adjusting for negative control should be 0 for all except M5. For each box, n = 300 estimates generated from 300 rounds of simulation under the given setting; the center line of the box represents the median; the lower and upper hinges of the box correspond to the first and third quartiles; the upper/lower whisker extends from the hinge to the largest/smallest value no further than 1.5 * inter-quartile range from the hinge. **c)** Histogram of the proportions of false discoveries observed among DEGs found by GSFA at LFSR < 0.05, across 300 simulations. The results of a simple *t*-test comparing targeted cells against negative control cells are included for comparison, with DEGs discovered at FDR < 0.05. Source data

**Extended Data Fig. 4:**
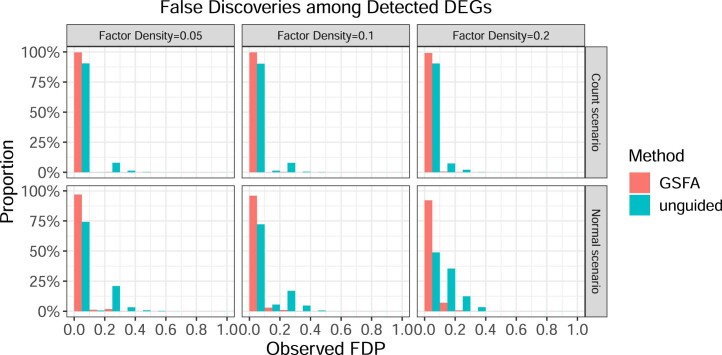
GSFA vs. two-step factor analysis in simulations. Shown are the false discovery proportions (FDP) of the DEGs detected by either method. Source data

**Extended Data Fig. 5:**
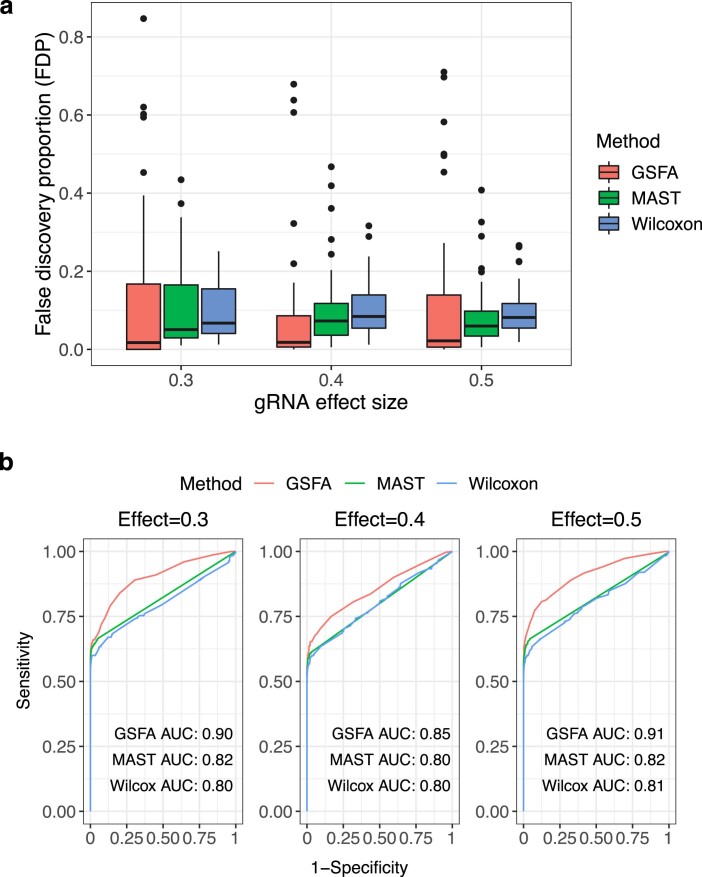
The performance of GSFA compared with MAST and Wilcoxon on the simulation dataset, where target genes of perturbation were chosen randomly. Each panel in the figure displays the results for three gRNAs with varying effect sizes, measured by standard deviations. The differential analysis is comparing each gRNA against cells perturbed by negative control gRNA. Panel **a)** shows the false discovery proportion (FDP) of GSFA, MAST, and Wilcoxon with effect size being 0.3, 0.4, and 0.5. For each box, n = 50 estimates generated from 50 rounds of simulation under the given setting; The centerline of a box represents the median; the lower and upper hinges of a box correspond to the first and third quartiles; the upper/lower whisker extends from the hinge to the largest/smallest value no further than 1.5 * inter-quartile range from the hinge. Panel **b)** shows the ROC of these three methods. Source data

Finally, we evaluated GSFA under different parameter settings. In one setting, we introduced a special ‘negative control’ perturbation and changed the effect sizes of the perturbations on factors to mimic the nonspecific effects of gRNA perturbation on gene expression (see Supplementary Table 6 for the effect-size matrix). GSFA adjusted the nonspecific effects, leading to accurate parameter estimation and calibrated LSFR (Extended Data Fig. 3). In another setting, we allowed each perturbation to affect multiple factors (Supplementary Table 7). We then compared GSFA with a two-step factor analysis procedure, where one first performs factor analysis on the expression data and then associates perturbations with factors. This type of procedure has been used in previous single-cell CRISPR screening data^31^. To use this procedure for DEG analysis, we defined the targets of a perturbation as the union of all genes loaded on the factors associated with this perturbation. We found that the false positive rates of the two-step procedure were substantially higher than the GSFA (Extended Data Fig. 4). In the last setting, we used a real scRNA-seq dataset and introduced gRNAs to perturb gene expression. Instead of using factors, we randomly chose genes as the targets of the gRNAs. This simulation also demonstrated that GSFA was better at detecting the target genes of gRNAs than existing methods (Extended Data Fig. 5).

Through these simulations, we demonstrated that GSFA is a powerful method to identify gene modules and specific genes affected by CRISPR perturbations.

### GSFA reveals the downstream effects of T cell regulators

We applied GSFA to a CROP-seq dataset of primary human CD8^+^ T cells^10^. The study targeted 20 genes involved in the T cell response, in stimulated and unstimulated T cells, and applied a clustering approach to characterize the effects of each perturbation. Although the authors found that perturbations of some genes were correlated with clusters characterized by T cell activation, many other genes were not associated with any cluster. Moreover, the study lacked systematic differential expression analysis to reveal specific genes affected by perturbations.

**Fig. 3:**
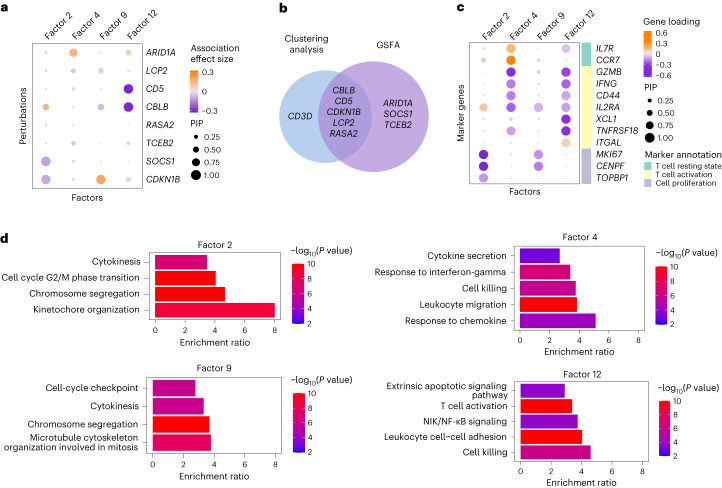
GSFA results of inferred factors from the analysis of CROP-seq data of primary CD8^+^ T cells. The results are based on stimulated T cells. **a**, Estimated effects of gene perturbations on selected factors inferred by GSFA. The size of a dot represents the PIP of the association; the color represents the effect size. **b**, Venn diagram of targets identified using the original clustering-based method versus GSFA. **c**, Loading of selected marker genes on selected factors. The size of a dot represents the gene PIP in a factor and the color represents the gene weight (magnitude of contribution) in a factor. **d**, Fold enrichment of selected GO ‘biological process’ gene sets significantly enriched (*q* < 0.05) in factors 2, 4, 9 and 12. Each bar is colored by −log_10_ *P* values from the overrepresentation test (an upper-tailed hypergeometric test), where overlap of a gene set with genes with a PIP > 0.95 in the factor was compared against that of all genes used in the GSFA. Source data

**Extended Data Fig. 6:**
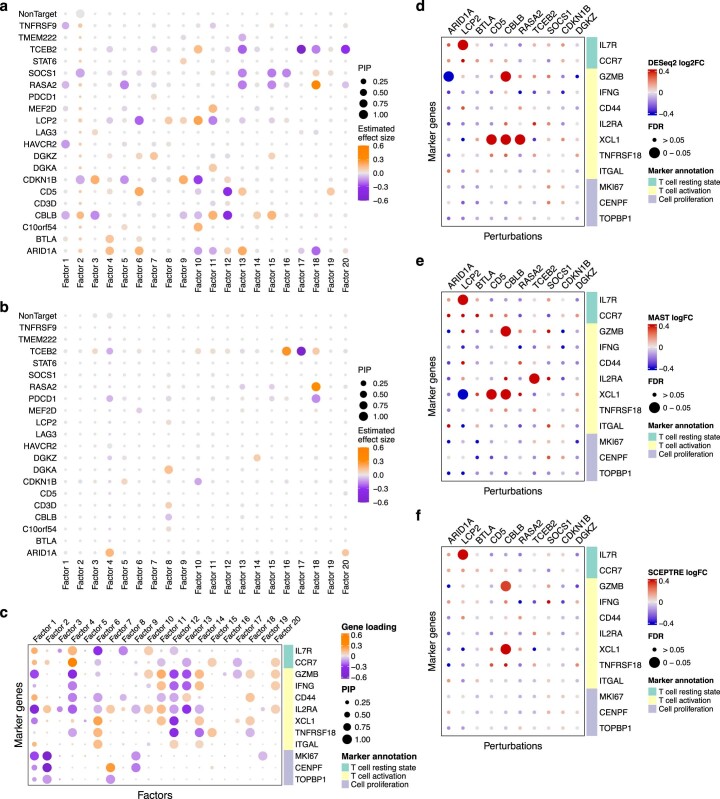
Additional GSFA results on CD8^+^ T cell CROP-seq dataset. **a)** Estimated effects of gene perturbations on all factors inferred by GSFA within stimulated T cells. The size of a dot represents the PIP of association; the color represents the effect size. **b)** Similar to a) but estimated within unstimulated T cells. **c)** Loading of selected marker genes on all factors. The size of a dot represents the gene PIP in a factor and the color represents the gene weight (magnitude of contribution) in a factor. **d–f)** Estimated effects of perturbations on marker genes in stimulated T cells with DESeq2 (d), MAST (e), and SCEPTRE (f). Sizes of the dots represent FDR bins; colors of the dots represent the DESeq2 log_2_ fold change estimates, the MAST log fold change estimates, and the SCEPTER log fold change estimates, respectively. Source data

When applying GSFA, we allowed perturbations to have different effects on factors in stimulated and unstimulated cells (Methods). We ran GSFA with 20 factors and verified that the results were generally robust to the number of factors (Supplementary Figs. 4 and 5). We found 24 associations (PIP > 0.95) between perturbations and factors in stimulated cells that involved eight gRNA-targeted genes (Fig. 3a for a subset of factors; full results in Extended Data Fig. 6a). Among these genes, the effects of *ARID1A*, *SOCS1* and *TCEB2* were undetected by clustering analysis in the original study (Fig. 3b). As expected, only three pairs of associations were detected at PIP > 0.95 in unstimulated cells (Extended Data Fig. 6b). We also confirmed, with permutation analysis, that the full GSFA results, including the inferred perturbation effects and gene loading, were calibrated (Supplementary Fig. 6a–c). Altogether, these results highlight the power of GSFA to detect broad effects of target genes on the latent factors.

For comparison, we also ran the model-based understanding of single-cell CRISPR screening (MUSIC) method^31^ to discover latent factors. MUSIC first performs topic models, a technique related to factor analysis, on the expression data; it then correlates the inferred factors with genetic perturbations across cells. Unexpectedly, almost all the perturbations correlated with all 20 topics discovered by MUSIC (Supplementary Fig. 7). These nonspecific findings made it difficult to understand the functions of the perturbed genes, so we did not pursue this analysis further.

To characterize the latent factors from the GSFA, we inspected the weights of canonical marker genes (Supplementary Table 1 and Extended Data Fig. 6c) and performed GO enrichment analysis of genes loaded on the factors (Supplementary Table 2). For example, factors 2 and 9 have negative weights for the cell proliferation markers *MKI67*, *TOPBP1* and *CENPF* (Fig. 3c), and are enriched for GO terms related to cell cycle and division (Fig. 3d). Factors 4 and 12 are associated with markers of T cell activation or resting states (Fig. 3c) and are enriched for GO terms related to immune responses (Fig. 3d). Together, these results show that the latent factors discovered by GSFA represent cellular processes.

**Fig. 4:**
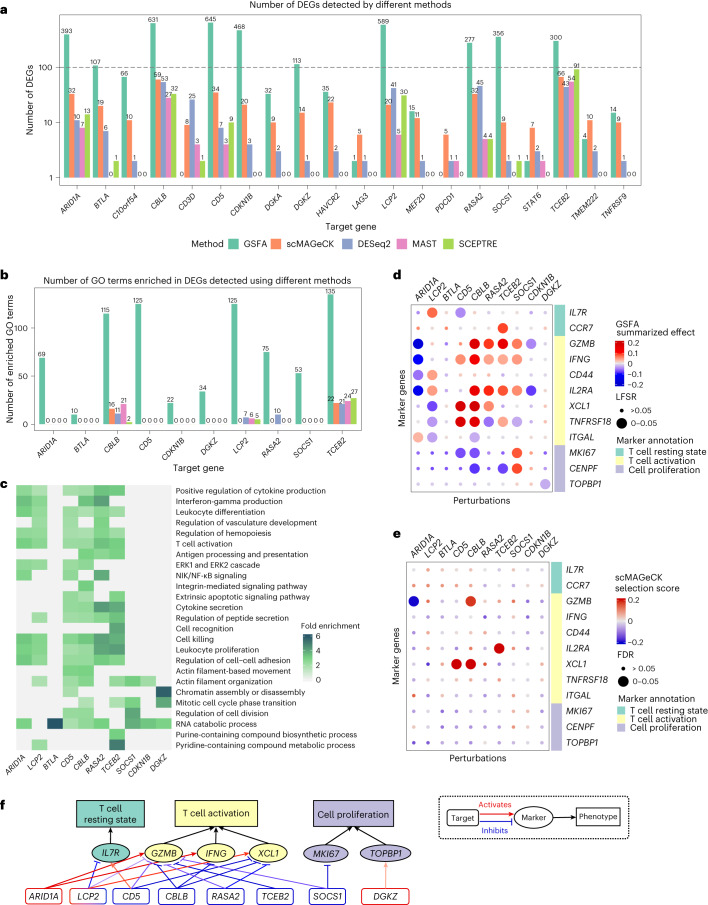
GSFA results of the effects of genetic perturbation on gene expression in CD8^+^ T cell data. Results are based on stimulated CD8^+^ T cells. **a**, Number of DEGs detected under all perturbations using four different methods. The *y* axis is log-scaled and the bar height corresponds to count +1 (as the number of DEGs could be 0); the exact numbers of DEGs are labeled on top of the bars. The detection threshold for DEGs is LFSR < 0.05 for GSFA and FDR < 0.05 for all other methods. **b**, Number of GO Slim ‘**b**iological process’ terms enriched in DEGs detected using different methods. **c**, Heatmap of selected GO ‘biological process’ terms and their fold enrichment in DEGs (LFSR < 0.05) detected using GSFA under different perturbations. **d**, GSFA estimated the effects of perturbations on marker genes in stimulated T cells. The sizes of the dots represent LFSR bins; the colors of the dots represent the summarized effect sizes. **e**, scMAGeCK estimated effects of perturbations on marker genes in stimulated T cells. The sizes of the dots represent the FDR bins; the colors of the dots represent the scMAGeCK selection scores. **f**, A target–marker–phenotype regulatory network summarizing the GSFA results. Significant (LFSR < 0.05) regulatory relationships between target and marker genes are represented by the colored arrows, with the red sharp arrows indicating positive regulation of marker genes by the target genes, and the blue blunt arrows indicating negative regulation. The darkness of the color represents the relative effect magnitude. Note that the effect directions here are the opposite of the perturbation effects. Source data

We note that one perturbation may affect multiple factors representing related processes. For instance, *CDKN1B* perturbation is associated with two cell cycle-related factors with opposite signs (factors 2 and 9; Fig. 3a,c). This makes it difficult to understand its effects. We thus used GSFA’s differential expression analysis (Fig. 1) to identify specific downstream genes of the perturbations. We also ran other DEG analysis methods for comparison, including MAST^30^, DESeq2 (ref. ^12^), edgeR-QLF^13^ and two methods tailored to single-cell CRISPR screening data, scMAGeCK-LR^32^ and SCEPTRE^33^. Among these methods, edgeR-QLF showed severe inflation in permuted data (Methods and Supplementary Fig. 6d–h); thus, it was excluded from further analysis. In stimulated T cells, GSFA detected more than 100 DEGs at an LFSR < 0.05 for ten gene targets, five of which (*ARID1A*, *BTLA*, *DGKZ*, *SOCS1* and *TCEB2*) were poorly characterized by clustering analysis in the original study^10^. Compared with other methods, GSFA consistently detected the most DEGs across these ten targets, sometimes ten times or more (Fig. 4a). Additionally, the DEGs of all ten target genes detected by GSFA were enriched for biologically relevant GO terms, while DEGs detected by other methods showed almost no GO enrichment (Fig. 4b,c).

**Extended Data Fig. 7:**
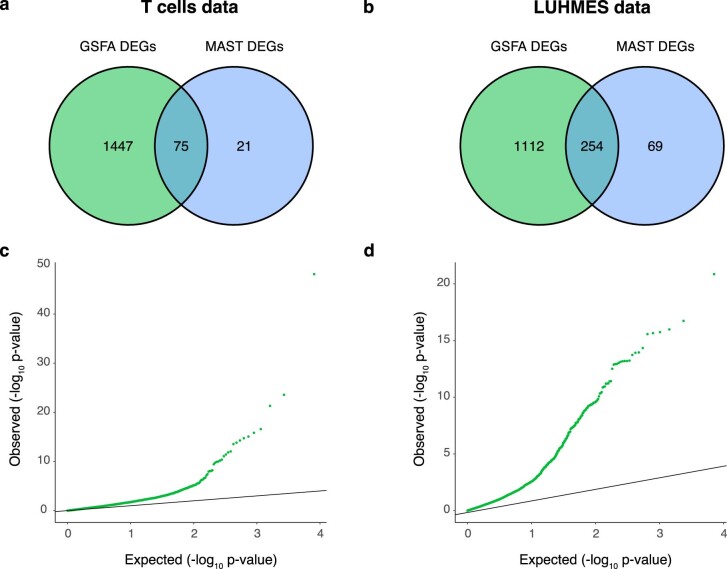
Assessing GSFA results for differential expression (DE) analysis using MAST. **a)**, **b)** Comparison of DEGs found by GSFA vs. MAST. a) T cells. b) LUHMES. **c)**, **d)** Quantile-quantile plot of p-values of differential expression estimated by MAST of GSFA detected DE genes, assuming a uniform(0,1) null distribution. c) T cells. d) LUHMES. Source data

We further compared the genes identified by GSFA and MAST, the method that detected the second highest number of DEGs. Most DEGs (>70%) from MAST were also discovered using GSFA (Extended Data Fig. 7a). Furthermore, a large proportion of GSFA-detected genes has low *P* values under MAST (Extended Data Fig. 7a). This suggests that the GSFA results were generally concordant with existing DEG analysis methods. By using information from coregulated genes, GSFA detected more DEGs whose significance fell below the statistical cutoff in the existing methods.

We next characterized the functions of the ten target genes by inspecting their effects on marker genes. GSFA revealed many effects of the target genes on the markers (Fig. 4d), many of which were missed by other methods (Fig. 4e for scMAGeCK; Extended Data Fig. 6d–f for the others). The estimated effects by GSFA largely agreed with the known functions of these genes. For instance, targeting of *CD5*, *CBLB* and *RASA2* had mostly positive effects on the markers of activated T cells, and negative or no effects on the markers of resting T cells (Fig. 4d), which is consistent with the functions of these genes as negative regulators of T cell activation^10^.

Our analysis provided insights on the functions of four (out of five) new genes, *ARID1A*, *DGKZ*, *SOCS1* and *TCEB2*, whose effects were poorly characterized in the original study (Fig. 3b). The effect of *TCEB2* perturbation on T cell markers is similar to those of other negative regulators of T cell responses, such as *CD5*. *DGKZ*-affected genes are enriched with GO terms related to the cell cycle (Fig. 4c) and *DGKZ* perturbation led to reduced expression of cell proliferation markers. These findings are consistent with the known role of *DGKZ* in regulating the cell cycle^34^. Targeting *SOCS1* has a strong effect on cell proliferation markers (Fig. 4d). Accordingly, several genes of the *SOCS* family have been reported to inhibit cell-cycle progression^34^. Targeting of *ARID1A*, a chromatin remodeler and potential tumor suppressor^35–37^, had strong negative effects on effector markers (Fig. 4d), suggesting its role as a positive regulator of T cell activation. Indeed, *ARID1A* mutations occur in many human cancer types and result in limited chromatin accessibility and downregulation of interferon-responsive genes, leading to poor tumor immunity^38^.

Collectively, GSFA revealed detailed transcriptional effects of genetic perturbations, including four genes largely missed by clustering or differential expression analysis with other tools. We constructed a regulatory network to summarize our major findings of the functions of nine target genes (Fig. 4f). Our results highlight the power of GSFA in revealing the detailed molecular effects of genetic perturbations in single-cell CRISPR screens.

### GSFA reveals the transcriptomic effects of autism risk genes

We next applied GSFA to CROP-seq data targeting 14 neurodevelopmental genes, including 13 autism risk genes, in LUHMES human neural progenitor cells^39^. After CRISPR targeting, cells were differentiated into postmitotic neurons and sequenced. The authors then projected cells onto a pseudotime trajectory, which approximates the progression of neuronal differentiation, and associated the perturbations with the pseudotime of cells. This analysis revealed the effects of several target genes on neuronal differentiation. However, it provided limited information on the molecular processes affected by the target genes other than pseudotime.

**Fig. 5:**
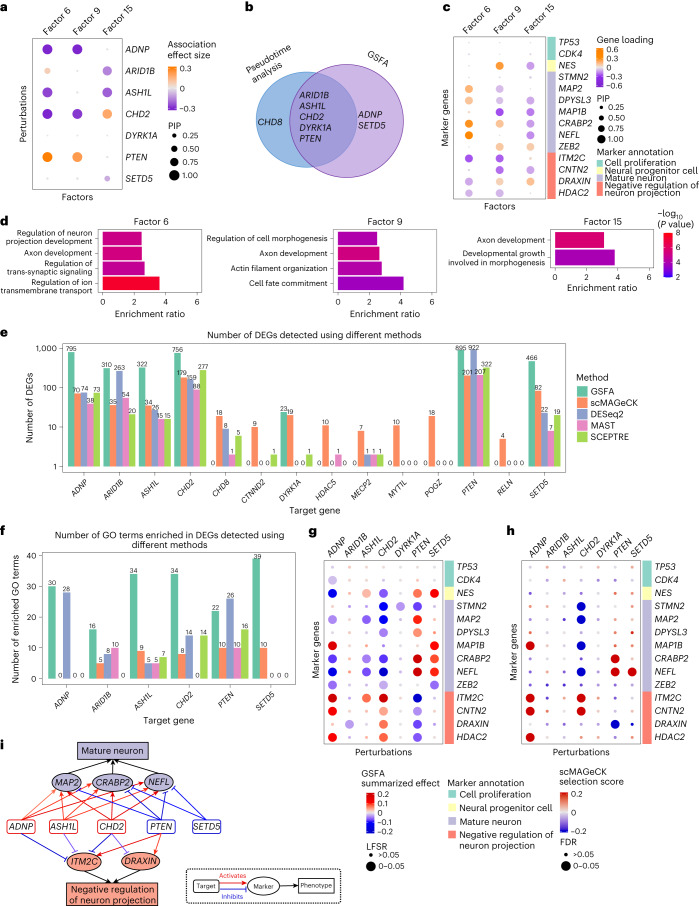
GSFA analysis of the CROP-seq data of LUHMES cells. **a**, Estimated effects of gene perturbations on selected factors inferred using GSFA. The size of a dot represents the PIP of association; the color represents the effect size. **b**, Venn diagram of targets identified from the original pseudotime association analysis versus from the GSFA. **c**, Loading of neuronal marker genes on selected factors. The size of a dot represents the gene PIP in a factor and the color represents the gene weight (magnitude of contribution) in a factor. **d**, Fold of enrichment of selected GO ‘biological process’ terms enriched in factors 4, 9 and 16 (*q* < 0.05). Each bar is colored using −log_10_ *P* values from the overrepresentation test (an upper-tailed hypergeometric test), where overlap of a gene set with genes with PIP > 0.95 in the factor was compared against that of all genes used in the GSFA. **e**, Number of DEGs detected under all perturbations using four different methods. The *y* axis is log-scaled and the bar height corresponds to count +1 (as the number of DEGs could be 0); the exact number of DEGs is labeled above the bars. The detection threshold for DEGs is LFSR < 0.05 for GSFA and FDR < 0.05 for all other methods. **f**, Number of GO Slim ‘biological process’ terms enriched in DEGs detected using different methods. **g**, GSFA estimated effects of perturbations on marker genes. The sizes of the dots represent the LFSR bins; the colors of the dots represent the summarized effect sizes. **h**, scMAGeCK estimated effects of perturbations on marker genes. The sizes of the dots represent the FDR bins; the colors of the dots represent the scMAGeCK selection scores. **i**, Target–marker–phenotype regulatory network summarizing the GSFA results. Significant (LFSR < 0.05) regulatory relationships between target and marker genes are represented by the colored arrows, with the red sharp arrows indicating positive regulation of marker genes by target genes, and the blue blunt arrows indicating negative regulation. The darkness of the color represents the relative magnitude of effect. Note that the direction of regulation is the opposite of the perturbation effect. Source data

**Extended Data Fig. 8:**
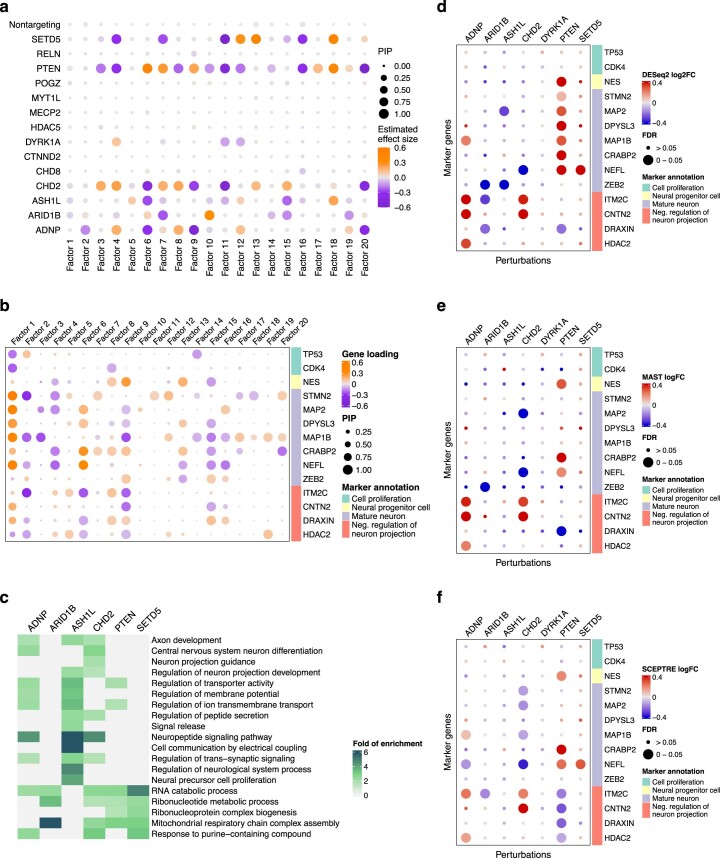
Additional GSFA results on LUHMES CROP-seq dataset. **a)** Estimated effects of gene perturbations on all factors inferred by GSFA. The size of a dot represents the PIP of association; the color represents the effect size. **b)** Loading of neuronal marker genes on all factors. The size of a dot represents the gene PIP in a factor and the color represents the gene weight (magnitude of contribution) in a factor. **c)** Heatmap of selected GO ‘biological process’ terms and their folds of enrichment in DEGs detected by GSFA (LFSR < 0.05). **d–f)** Estimated effects of perturbations on marker genes in LUHMES with DESeq2 (d), MAST (e), and SCEPTRE (f). Sizes of the dots represent FDR bins; colors of the dots represent the DESeq2 log_2_ fold change estimates, the MAST log fold change estimates, and the SCEPTER log fold change estimates, respectively. Source data

After applying GSFA to this dataset, we first confirmed that GSFA did not produce false positive findings in permutations (Supplementary Fig. 8). We found significant effects (PIP > 0.95) of six target genes, including *ADNP*, *ARID1B*, *ASH1L*, *CHD2*, *PTEN* and *SETD5*, on at least one out of 20 latent factors (Fig. 5a for a subset of factors; Extended Data Fig. 8a for the full results). Among the six genes, the transcriptomic effects of *ADNP* and *SETD5* were missed in the original pseudotime-based analysis (Fig. 5b). We characterized these factors by inspecting the weights of neuronal markers (Supplementary Table 3 and Extended Data Fig. 8b) and GO enrichment analysis (Supplementary Table 4). In factor 6, for example, the markers of mature neurons such as *MAP2* and *NEFL* had positive weights, while negative regulators of neuron projection, such as *ITM2C*, had negative weights (Fig. 5c), suggesting that factor 6 is positively associated with neuronal maturation. Indeed, factor 6 is significantly enriched for gene sets involved in neuronal development (Fig. 5d). Factors 9 and 15, similarly, showed loadings of neuronal markers and were enriched for relevant GO terms (Fig. 5c,d).

We next identified the individual genes affected by the perturbations. GSFA detected more than 100 DEGs at LFSR < 0.05 for the same six gene targets (Fig. 5e). Compared with other differential expression analysis methods, GSFA detected the most DEGs for five out of six gene targets (Fig. 5e). Furthermore, DEGs detected using GSFA were enriched for the most GO terms across almost all targets (Fig. 5f), many of which are related to neuronal development or neural signaling (Extended Data Fig. 8c). Like our analysis of the T cell data, we also compared the actual DEGs found using GSFA and other methods and found general concordance (Extended Data Fig. 7b).

To understand the functions of these six target genes, we examined their effects on marker genes for neuron maturation and differentiation. GSFA uncovered perturbation effects on several marker genes across all targets except *ARID1B* (Fig. 5g), while other methods detected fewer differentially expressed markers (Fig. 5h for scMAGeCK; Extended Data Fig. 8d–f for DESeq2, MAST and SCEPTRE). GSFA-estimated effects largely validated the known functions of these genes on neuronal maturation phenotypes^39^. Targeting of *ASH1L* and *CHD2* had mostly negative effects on mature neuronal markers and positive effects on negative regulators of neuron projection (Fig. 5g), indicating delayed neuron maturation by the repression of these genes. Knockdown of *PTEN* showed the opposite effects, suggesting its opposite role on neuronal differentiation.

Two genes, *ADNP* and *SETD5*, were missed in the pseudotime-based analysis in the original study (Fig. 5b). The estimated effects of these genes on neuronal markers by GSFA suggested that repression of *ADNP* would lead to delayed neuronal differentiation, whereas *SETD5* repression would have the opposite effect (Fig. 5g). These predictions are consistent with the experimental finding of *ADNP*^39^ and with the finding that *SETD5* knockdown increases the proliferation of cortical progenitor cells and neural stem cells^40^.

In conclusion, GSFA allowed us to characterize the transcriptional effects of six autism spectrum disorder risk genes, including *ADNP* and *SETD5*, whose effects were largely missed in the original study. While GSFA missed the effect of *CHD8* (Fig. 5b), we noticed that all the existing DEG methods also largely missed its effect (Fig. 5e). We summarized the inferred target effects of GSFA on selected marker genes and affected cellular processes in a gene regulatory network (Fig. 5i).

## Discussion

Single-cell CRISPR screening technologies have enabled efficient readouts of transcriptome-level effects of multiple genetic perturbations in a single experiment. These technologies offer great opportunities, but also challenges for effective data analysis. We presented GSFA to address these challenges. GSFA identifies gene modules that respond to genetic perturbations; by summarizing the information from these factors, it infers the effects of perturbations on downstream genes. When applied to two CROP-seq datasets, the GSFA results shed light on the molecular mechanisms of regulators of T cell activation and neuronal differentiation, respectively.

The GSFA model is built on factor analysis^41,42^ and is related to existing factor models. In particular, one could perform a factor analysis first on expression data and then correlate the genetic perturbations with the inferred factors^31^. Compared with this two-step approach, GSFA has several advantages. When inferring expression factors, GSFA uses the genetic perturbation as a prior to improve the estimation of the factors (hence ‘guided’ in the name of the methos; Methods). GSFA also offers an important advantage when a perturbation affects multiple factors. With each topic representing a somewhat different process, it is difficult to interpret the possible effects of perturbations. GSFA solves the challenge of the two-step procedure by synthesizing the effects of perturbation over all factors and showed better control of false discoveries in simulations. GSFA is also related to a class of factor models in the statistics literature, sometimes called supervised factor analysis, where the factors depend on covariates of the samples^43–45^. These models can help improve the estimation of latent factors and have been proposed in bulk gene expression data analysis^46^, where samples have different characteristics or experimental conditions. Nevertheless, existing covariate-dependent factor models were designed only for factor inference and do not provide estimates of the effects of covariates (perturbations in our case) for specific genes.

GSFA is a general statistical model and in principle can be applied to any single-cell CRISPR screening dataset. In practice, it is better suited for some settings than others. GSFA is most powerful when the perturbations have large effect sizes, affecting the expression of many genes. In some experiments^11^, researchers targeted noncoding elements, whose effects may be small and limited to the expression of nearby genes. GSFA may not be beneficial in such cases. Another key consideration is the multiplicity of infection (MOI) in experiments. We have applied GSFA to the low MOI setting, where a cell usually contains at most one gRNA. The high MOI setting may pose unique challenges. For example, multiple perturbations in a cell may interact nonadditively, and technical confounders may lead to false discoveries^33^. Additional work needs to be done to evaluate GSFA in the high MOI setting.

GSFA can be further improved along several directions. GSFA does not directly model read counts and instead uses deviance residuals converted from count data. We noticed that the LFSRs from differential expression analysis can be modestly inflated at high factor density (under *π* = 0.2). Directly modeling read counts may improve the calibration of GSFA. Another limitation of GSFA is that we assume that genetic perturbations affect downstream genes only through factors. It is possible that the factors may not fully capture the transcriptional effects; thus, it may be desirable to add ‘direct effect’ terms, where perturbations directly affect the expression of a gene without acting on any factors. Finally, GSFA uses Gibbs sampling for inference; replacing this with a more efficient algorithm, such as variational approximation, may improve computational efficiency.

In conclusion, single-cell CRISPR screening is a promising technology, yet data analysis from such experiments is challenging. GSFA offers a powerful new analysis framework, allowing researchers to better realize the potential of single-cell screening technology.

## Methods

### GSFA model

The input data of GSFA consist of a gene expression matrix *Y*_*N* × *P*_ with *N* cells and *P* genes, and a perturbation matrix ${G}_{N\times M}$ with *N* cells and *M* types of genetic perturbations. In all our analyses, the perturbation matrix was binary, that is, *G*_*im*_ $=1$ if cell *i* has the *m*-th type of perturbation and 0 otherwise, but this is not strictly required by the model; for example, *G* might represent the dosage of genetic perturbations. The GSFA model has two main parts: (1) a sparse factor analysis model that decomposes the expression matrix *Y* into a factor matrix ${Z}_{N\times K}$, where *K* is the number of factors, and a sparse gene weight matrix ${W}_{P\times K}$; and (2) a multivariate linear model that correlates the factor matrix *Z* with the perturbation matrix *G*. Let *i*, *j* and *k* be indices of cells, genes and factors, respectively:

1 $$Y=Z{W}^{{\rm{T}}}+E,{E}_{{ij}}\sim N(0,{\boldsymbol{\psi }}_{j})$$

2 $$Z=G{\boldsymbol{\beta }}+\Phi ,{\phi }_{ik}\mathop{\sim }\limits N(0,1)$$

*E* is an *N* × *P* residual matrix with gene-specific variances stored in a *P* vector **ψ**, *β* is an *M* × *K* matrix of perturbation effects on factors, ${\boldsymbol{\varPhi }}$ is an *N* × *M* residual matrix with variance 1 and W^T^ is the transpose of W. Compared with standard factor analysis, our model assumes that the latent factor *Z* also depends on the additional covariates *G*; hence, our model is a form of ‘guided’ factor analysis.

We assume that each perturbation affects only a small number of factors, so we impose a ‘spike-and-slab’ prior on the effect of perturbation *m* (1 ≤ *m* ≤ *M*) on factor *k* (1 ≤ *k* ≤ *K*):

3 $${\beta }_{{mk}}\sim {p}_{m}N\left(0,{d}_{m}^{\,2}\right)+\left(1-{p}_{m}\right){\delta }_{0}$$

where *δ*_0_ is delta function, *p*_*m*_ denotes the proportion of factors affected by perturbation *m* and *d*_*m*_ the prior variance of the effect sizes of *m*.

To limit the number of genes contributing to a factor and facilitate the biological interpretation of factors, we also imposed a sparse prior on the gene weights. We found in our simulations and real data analysis that, when analyzing count data, the standard spike-and-slab prior is sometimes insufficient to impose sparsity (Supplementary Note 3.1). We think this is due to a well-known problem in count-based RNA-seq data analysis: because the total read count in a sample is fixed, activation of some genes indirectly reduces the read counts in all other genes, resulting in weakly correlated expression across many genes. Thus, even when a factor affects only a small set of genes, it may appear to be correlated with many other genes, making it hard to infer sparse factors. So we chose a ‘normal mixture’ prior. This prior assumes that the gene weights in a factor come from a mixture of two normal distributions with mean 0 but different variances. The difference with the spike-and-slab prior is that the ‘background’ component is not necessarily *δ*_0_, but rather a distribution with small effects. The prior weight of gene *j* in the factor *k* follows:

4 $${W}_{{jk}}\sim {\pi }_{k}N\left(0,{\sigma }_{k}^{2}\right)+\left(1-{\pi }_{k}\right)N\left(0,{\sigma }_{k}^{2}{c}_{k}^{2}\right),0 < {c}_{k} < 1$$

where *π*_*k*_ represents the proportion of genes affected by the factor *k* (the ‘foreground’ part), ${\sigma }_{k}^{2}$ the prior effect size variance of factor *k* and *c*_*k*_ a scale parameter controlling the relative size of the foreground and background effects.

The prior distributions for other parameters in the model are specified in Supplementary Note 1.1.

### GSFA model inference

We inferred the parameters in GSFA using Gibbs sampling, a Markov chain Monte Carlo (MCMC) algorithm that obtains a sequence of approximate samples from their posterior distribution given the observed data. Gibbs sampling is an attractive choice because the conditional distributions of the main parameters (*β* and *W*) and latent variables (*Z*) have analytical forms. To see this, we first considered the conditional distribution of *W*, given data and all other parameters and variables, *P*(*W*|*Y*, *G*, *Z*, *β*). (For simplicity, we dropped the hyperparameters and parameters related to the error terms.) It is easy to see that given *Z*, *W* does not depend on *G* and *β*, so we have:

5 $$P\left(W|Y,G,Z,{\boldsymbol{{\beta} }}\right)=P\left(W|Y,Z\,\right)$$

The problem now becomes multivariate linear regression, *Y* = *ZW*^T^ + *E*, where *W* follows a spike-and-slab prior. This is a well-studied problem in the statistics literature^47,48^. Similarly, we can see that the conditional distribution of *β* is given by:

6 $$P\left(\beta |Y,G,Z,W\right)=P\left(\beta |G,Z\right)$$

Again, this reduces to a regression problem *Z* = *Gβ* + *Φ*, where *β* follows the normal-mixture prior. Finally, the conditional distribution of *Z* is given by:

7 $$P\left(Z{\rm{|}}Y,G,W,\beta \right)\propto P\left(Z{\rm{|}}G,{\boldsymbol{\beta }}\right) P\left(Y{\rm{|}}Z,W\,\right)$$

This is also a regression problem *Y* =*ZW*^T^ + *E*, where *Z* represents the unknown coefficients, with a normal prior, *Z*_*i*_ ≈ $N$(*G*_*i*_*β*, *I*), for the sample *i* (1 ≤ I ≤ *N*). We now see that the posterior of *Z* not only depends on the gene expression matrix *Y*, but also the perturbations *G*. In other words, the perturbations impose a prior on *Z*, hence ‘guiding’ the inference of *Z* in a certain sense.

To facilitate computation, we also introduced two latent binary matrices, *F*_*P* × *K*_ and ${{\boldsymbol{\gamma }}}_{M\times K}$, to indicate which distribution the corresponding parameters in *W* and *β* come from. The joint prior distribution of *W* and *F* follows:

8 $$\begin{array}{l}P\left({F}_{{jk}},{W}_{{jk}}\right) \\ =P\left({W}_{{jk}}{\rm{|}}{F}_{{jk}}\right)P\left({F}_{{jk}}\right)=N\Big({W}_{{jk}}{\rm{;}}0,{\sigma }_{k}^{2}\left[{F}_{{jk}}+\left(1-{F}_{{jk}}\Big){c}_{k}^{2}\right]\right) {\pi }_{k}^{{F}_{{jk}}}{\left(1-{\pi }_{k}\right)}^{1-{F}_{{jk}}}\end{array}$$

The joint prior distribution of *β* and *γ* can then be written as:

9 $$P\left({\beta }_{{mk}}{\rm{|}}{\gamma }_{{mk}}=0\right)P\left({\gamma }_{{mk}}=0\right)=1-{p}_{m}$$

10 $$P\left({\beta }_{{mk}}{\rm{|}}{\gamma }_{{mk}}=1\right)P\left({\gamma }_{{mk}}=1\right)={p}_{m}N\left({\beta }_{{mk}}{\rm{;}}0,{d}_{m}^{\,2}\right)$$

The details of the Gibbs sampling steps are described in Supplementary Note 1.2.

Unless mentioned otherwise, for all the datasets in the study, we ran the MCMC chain for 3,000 iterations and used the last 1,000 iterations to obtain the posterior samples of the parameters.

The posterior distribution allowed us to summarize the probabilities that some effects are nonzero. Specifically, the posterior mean of ${\gamma }_{{mk}}$ gives the PIP of ${\beta }_{{mk}}$, that is, the probability of ${\beta }_{{mk}}$ being nonzero as:

11 $${\rm{PIP}}\left({\beta }_{{mk}}\right):=\text{Pr}\left({\beta }_{{mk}}\ne 0\text{|data}\right)=\text{Pr}\left({\gamma }_{{mk}}=1\text{|data}\right)$$

Similarly, the posterior mean of ${F}_{{jk}}$ gives the PIP of ${W}_{{jk}}$ defined as the probability of ${W}_{{jk}}$ coming from the ‘foreground’ normal d istribution-given data:

12 $${\rm{PIP}}(W_{jk}) := {\rm{Pr}} (W_{jk}\, {\rm{comes}}\ {\rm{from}}\ {\rm{larger}}\ {\rm{effect}}|{\rm{data}}) = {\rm{Pr}} (F_{jk} = 1|{\rm{data}}) .$$

### Summarizing the effects of genetic perturbations on individual genes

While the effects of genetic perturbations are formulated in terms of factors under the GSFA, the model allows us to infer the effects on individual genes. This is similar to the commonly used differential gene expression analysis, where the expression of genes in cells with certain perturbation are compared with those without it. Under our model, the effect of perturbation *m* on the expression of gene *j* is mediated through one or more factors. The total effect, denoted as ${\theta }_{{mj}}$, is then given by the sum of *K*-mediated effects:

13 $${\theta }_{{mj}}=\sum _{k}{\beta }_{{mk}}{W}_{{jk}}$$

To sample the posterior distribution of ${\theta }_{{mj}}$, we use the posterior samples of ${\beta }_{{mk}}$ and ${W}_{{jk}}$:

14 $${\theta }_{{mj}}^{\left(t\right)}=\mathop{\sum }\limits_{k=1}^{K}{\beta }_{{mk}}^{\left(t\right)}{W}_{{jk}}^{\,\left(t\right)}{F}_{{jk}}^{\,\left(t\right)}$$

where superscript (*t*) denotes the *t*-th posterior sample. While the posterior distribution of ${\theta }_{{mj}}$ contains all the information we have, in practice, it is simpler to use a single summary of how likely ${\theta }_{{mj}}$ is nonzero. To do this, we used the LFSR, a metric that is analogous to LFDR but reflects confidence in the sign of effect rather than in the effect being nonzero^27^. LSFR has some benefits over the commonly used FDR approach, and is in fact more conservative than LFDR. The LFSR of the perturbation effect on individual genes, ${\theta }_{{mj}}$, is given by:

15 $${\mathrm{LFSR}}\left({\theta }_{{mj}}\right)=\min \Big\{\Pr \left({\theta }_{{mj}}^{\left(t\right)}\ge 0{\rm{|}}{\mathrm{data}}\right),\Pr \left({\theta }_{{mj}}^{\left(t\right)}\le 0{\rm{|}}{\mathrm{data}}\right)\Big\}$$

By thresholding the LFSR, we can obtain significant DEGs under each perturbation. In practice, the threshold is LFSR < 0.05.

### Applying GSFA to single-cell CRISPR screening data

When applied to real data, GSFA first transforms the count data using deviance residual transformation (Supplementary Note 2.1). GSFA also allows us to adjust for the nonspecific effects of gRNAs through negative control gRNAs. Briefly, the effect of a perturbation *m* on the factor *k*, ${\beta }_{{mk}}$, is adjusted as ${\beta }_{{mk}}^{{\prime} }={\beta }_{{mk}}-{\beta }_{0k}$, where ${\beta }_{0k}$ is the effect of negative control gRNAs on the factor *k*. The total effect of perturbation *m* on gene *j* is now ${\theta }_{{mj}}^{{\prime} }={\sum }_{k}{\beta }_{{mk}}^{{\prime} }{W}_{{jk}}$. With these adjustments, we can still obtain the posterior samples of the perturbation-to-factor and perturbation-to-gene effects, and do the LFSR control as before. We verified that this procedure corrects for nonspecific effects of gRNAs in simulations, and used it in our analysis of both real datasets.

For more information about GSFA implementation and running time, see Supplementary Note 5 and Supplementary Table 5.

### Alternative DGE methods

For comparison, we applied the following DGE methods to simulated or real data: (1) two-sided Welch’s *t*-test^29^ using the t.test() function in the R base package stats; (2) edgeR-QLF^13^using the glmQLFit() and glmQLFTest() functions in the R package edgeR v.3.32.1; (3) DESeq2 (ref. ^12^) using the DESeq() function in the R package DESeq2 v.1.30.1; (4) MAST^30^, a statistical method tailored for scRNA-seq data, using the zlm() and lrTest() functions in the R package MAST v.1.16.0; (5) scMAGeCK-LR^32^, a linear regression-based approach tailored to single-cell CRISPR screening data, using the scmageck_lr() function in the R package scMAGeCK v.1.2.0. We did not include scMAGeCK-RRA because it is not designed to test all genes^32^; (6) SCEPTRE^33^, a statistical method that analyzes single-cell CRISPR screens via conditional resampling, using the run_sceptre_high_moi() function in the R package sceptre v.0.1.0.

### Simulation study

We simulated single-cell CRISPR screen data using the GSFA model with either continuous gene expression levels or discrete gene count data as the output. We simulated under *N* = 4,000 cells, *P* = 6,000 genes, *M* = 6 types of perturbations and *K* = 10 underlying factors: (1) normal model. Continuous gene expression levels generated under the following model:

16 $${G}_{im}\mathop{\sim }{\rm{Bern}}(0.05),{\phi }_{ik}\mathop{\sim }N(0,1)\to Z=G{\boldsymbol{\beta }}+\Phi$$

17 $${W}_{jk}\mathop{\sim }\pi N(0,0.5)+(1-\pi ){\delta }_{0},{E}_{ij}\mathop{\sim }N(0,1)\to Y=Z{W}^{{\mathrm{T}}}+E$$

where *π* represents the proportion of genes loaded on any factor and varies from 0.05, 0.1 to 0.2 under different simulation scenarios; (2) count model. To sample the read count data, we assumed that each cell had a library size or scaling factor *L*_*i*_, sampled from a normal distribution with mean 5 × 10^5^. The count of a gene *j* would then be sampled from a Poisson distribution with its mean determined by the continuous gene expression level *y*_*ij*_ and the scaling factor *L*_*i*_:

18 $${L}_{i}\sim N\left(5\times {10}^{5},{10}^{5}\right)\to {c}_{{ij}}\sim \text{Poisson}\left({L}_{i}\exp \left(1/5\times {10}^{5}+{y}_{{ij}}\right)\right)$$

The sampled counts are converted to deviance residuals (Supplementary Note 2.1), then centered and scaled so that each gene has variance 1 before being provided as input for GSFA.

We set the effect-size matrix *β* to the following form, so that each perturbation affects a distinct factor and the effect sizes vary from 0.1 to 0.6:

$${\boldsymbol{\beta }}=\left(\begin{array}{cccccccccc}0.1 & 0 & 0 & 0 & 0 & 0 & 0 & 0 & 0 & 0\\ 0 & 0.2 & 0 & 0 & 0 & 0 & 0 & 0 & 0 & 0\\ 0 & 0 & 0.3 & 0 & 0 & 0 & 0 & 0 & 0 & 0\\ 0 & 0 & 0 & 0.4 & 0 & 0 & 0 & 0 & 0 & 0\\ 0 & 0 & 0 & 0 & 0.5 & 0 & 0 & 0 & 0 & 0\\ 0 & 0 & 0 & 0 & 0 & 0.6 & 0 & 0 & 0 & 0\end{array}\right)$$

These effect sizes were chosen so that the perturbations explained about 0.2% to 8% of the total variance of each factor.

We generated 300 random datasets under each of the six scenarios (normal/count-based and *π* = 0.05, 0.1, 0.2) for GFSA analysis. For each dataset, Gibbs sampling was performed for 3,000 iterations and the posterior means of parameters were computed from the last 1,000 iterations.

We evaluated the results according to whether the factors were recovered and whether the genes affected by a perturbation were identified. Due to the interchangeability of factors in matrix factorization (equation (1)), we mapped each of the true factors to the GSFA inferred factor that was maximally correlated with using the absolute Pearson correlation. The correlations of the true and inferred factors were then assessed. To evaluate the identification of genes affected by perturbations, we defined the ground truth as the genes with nonzero weights on the factors affected by a perturbation.

We also evaluated GSFA under additional parameter settings. The first setting was designed to mimic the nonspecific effects of gRNAs. We added one perturbation as a negative control and allowed all perturbations to have a common effect on one factor (factor 5). The effect-size matrix is shown in Supplementary Table 6. The second setting mimicked a more complex relationship between perturbations and factors. Under this setting, each of six perturbations affected three out of ten factors. For simplicity, we used a common effect size of 0.4 for all perturbation effects (see Supplementary Table 7 for the effect-size matrix). In the last setting, we created simulation data using real scRNA-seq data without explicitly introducing latent factors (Supplementary Note 6). Details of how other methods were run in the simulations are also provided in Supplementary Note 6.

### GSFA analysis of the CD8^+^ T cell CROP-seq dataset

Raw cellranger outputs of the CD8^+^ T cell CROP-seq study^10^ were downloaded from the Gene Expression Omnibus (accession no. GSE119450). We merged resting and stimulated T cells from two donors using the R package Seurat v.4.0.1 (ref. ^49^). We first filtered cells that contained fewer than 500 expressed genes or more than 10% of the total read counts from mitochondrial genes, keeping 14,278 stimulated T cells and 10,677 unstimulated T cells. Next, we transformed the raw counts into deviance residuals for all genes in all cells, kept the top 6,000 genes ranked using deviance statistics (Supplementary Note 2.1), then regressed out the unique UMI count, library size and percentage of mitochondrial gene expression from the reduced deviance residual matrix. The resulting matrix was then scaled so that each gene had variance 1.

The gRNA perturbation data were binarized, with gRNAs targeting the same gene deemed as the same type of perturbation. The scaled gene expression and perturbation matrices were used as input for GSFA. To capture potentially different effects of CRISPR perturbation under resting and stimulated conditions, we used the modified GSFA model with two cell groups (Supplementary Note 3.2), stratifying all cells according to their stimulation states (unstimulated: 0, stimulated: 1). By inspecting how the percentage of gene expression explained varied with the number of latent factors, we chose 20 factors in our analysis (Supplementary Note 4 and Supplementary Fig. 4). We verified that the main results of the GSFA in terms of DEGs found for each perturbed gene were generally robust to the number of factors (Supplementary Fig. 5). Gibbs sampling was performed for 4,000 iterations and the posterior means of parameters were computed from the last 1,000 iterations.

We assessed the calibration of the GSFA results using permutation. We created ten permutation sets on the stimulated and unstimulated cells separately. In each permutation set, the cell labels were permuted independently of the perturbation conditions and GSFA was run on each of these datasets. The calibration was assessed in a few ways. We checked the distribution of PIPs of the perturbation effects on factors (*β*) and the distribution of LSFRs from the inferred perturbation to gene effects. We expected PIPs to be close to 0 and LSFRs close to 1 in the permutation results. We also assessed the empirical *P* values of the correlations between perturbations and inferred factors. Because we did not expect any correlation between the two under permutation, any deviation of *P* values from the null distribution would indicate that GSFA incorrectly borrowed information from perturbations to infer factors, a potential problem that would inflate the results.

### GSFA analysis of LUHMES CROP-seq dataset

Raw cellranger outputs of the LUHMES neural progenitor cell CROP-seq study^39^ were downloaded from the GEO (accession no. GSE142078). We merged all three batches of LUHMES CROP-seq raw data together using the R package Seurat v.4.0.1 (ref. ^49^), and filtered cells with a library size over 20,000 or more than 10% of the total read counts from mitochondrial genes, keeping 8,708 cells. Similarly, we transformed the raw count matrix into a reduced deviance residual matrix with the top 6,000 genes ranked according to the deviance residual (Supplementary Note 2.1). Differences in experimental batch, unique UMI count, library size and percentage of mitochondrial gene expression were all regressed out. Running the GSFA was the same as before, except that there was only one cell group and Gibbs sampling was run for 3,000 iterations. We also verified that it was reasonable to use 20 factors and that the results were insensitive to this number (Supplementary Figs. 4 and 5). We then assessed the results of the calibration of GSFA in the same way as we did with the T cell analysis.

### Running alternative methods on CD8^+^ T cell and LUHMES CROP-seq data

For both stimulated T cells and LUHMES CROP-seq data, we performed alternative DGE analyses for comparison. We applied edgeR-QLF^13^, DESeq2 (ref. ^12^) and MAST^30^ directly to the scRNA-seq raw count data, contrasting cells with each perturbation from those without, for all the genes selected for GSFA. For the LUHMES dataset, the experimental batch was included as one of the covariates in these three tests. We also applied scMAGeCK-LR^32^ to the transformed and corrected CROP-seq data (described above).

We applied SCEPTRE^33^ (using the R package sceptre v.0.1.0) to the scRNA-seq raw count data. We included the unique UMI count, library size and percentage of mitochondrial gene expression as covariates in the stimulated T cell data. For the LUHMES dataset, experimental batch was also included as one of the covariates. We used the default parameter settings in the run_sceptre_high_moi() function under the two-sided test setting.

For all these methods, FDR was computed using the Benjamini–Hochberg procedure for genes under each perturbation; significant DEGs were obtained under an FDR cutoff of 0.05.

To assess the calibration of the differential expression test *P* values from these methods, we carried out permutation tests for each DGE method by randomly shuffling the cell labels independent of the perturbation conditions. For the T cell dataset, shuffling occurred within the stimulated cells. We generated ten permuted datasets and performed the DGE methods in the same way as before.

We applied MUSIC^31^ (using the R package MUSIC v.1.0) directly to the scRNA-seq raw count data, following its own data preprocessing procedure. We varied the number of topics from 4, 5, 6 up to 20 topics, and observed similar patterns. We finally chose 20 topics so that the results could be comparable to the GSFA (fitted using 20 factors). To obtain the perturbation effects on inferred topics, we adapted the MUSIC’s Diff_topic_distri() function to obtain the *t*-test statistics and then further computed empirical *P* values by generating 10,000 permutations of the perturbation conditions.

### GO enrichment analysis

GO overrepresentation analyses were performed using the WebGestaltR() function in the R package WebGestaltR v.0.4.4 (ref. ^50^) with default parameters and the functional category for enrichment analysis set to the GO Slim ‘biological process’ category (geneontology_Biological_Process_noRedundant). To interpret the GSFA-inferred factors (gene modules), genes with weight PIP > 0.95 were treated as the foreground, while all genes used in the GSFA were treated as the background in the overrepresentation analysis. To interpret DEGs discovered under each perturbation using GSFA or other DGE methods, genes with an LSFR < 0.05 (or FDR < 0.05) were treated as the foreground, while all genes evaluated were treated as the background in the overrepresentation analysis.

### Reporting summary

Further information on research design is available in the Nature Portfolio Reporting Summary linked to this article.

## Online content

Any methods, additional references, Nature Portfolio reporting summaries, source data, extended data, supplementary information, acknowledgements, peer review information; details of author contributions and competing interests; and statements of data and code availability are available at 10.1038/s41592-023-02017-4.

### Supplementary information

Supplementary Information Supplementary Figs. 1–8, Tables 1–7 and Notes.

Reporting Summary

Supplementary Tables 2 and 4 Supplementary Table 2. Full GO enrichment results in T cell GSFA factors. Table 4. Full GO enrichment results in LUHMES GSFA factors

### Source data

Source Data Fig. 2 Statistical source data.

Source Data Fig. 3 Statistical source data.

Source Data Fig. 4 Statistical source data.

Source Data Fig. 5 Statistical source data.

Source Data Extended Data Fig. 1 Statistical source data.

Source Data Extended Data Fig. 2 Statistical source data.

Source Data Extended Data Fig. 3 Statistical source data.

Source Data Extended Data Fig. 4 Statistical source data.

Source Data Extended Data Fig. 5 Statistical source data.

Source Data Extended Data Fig. 6 Statistical source data.

Source Data Extended Data Fig. 7 Statistical source data.

Source Data Extended Data Fig. 8 Statistical source data.

## Data Availability

Both CROP-seq datasets used in this study are publicly available and were downloaded from the GEO (accession nos. GSE119450 and GSE142078). Source data are provided with this paper.

## Code availability

The R package implementing the GSFA is freely available at https://github.com/xinhe-lab/GSFA. The source code used in our study is deposited at https://github.com/xinhe-lab/GSFA_paper.
