# Supplementary material for: A new Bayesian factor analysis method improves detection of genes and biological processes affected by perturbations in single-cell CRISPR screening
Source: Nat Methods. 2023 Sep 28;20(11):1693–703. doi: 10.1038/s41592-023-02017-4 (PMC10630124; doi:10.1038/s41592-023-02017-4)
Supplement: Supplementary file 1 — Supplementary Figs. 1–8, Tables 1–7 and Notes. [file 41592_2023_2017_MOESM1_ESM.pdf]

nature methods

Article

https://doi.org/10.1038/s41592-023-02017-4

A new Bayesian factor analysis method improves detection of genes and biological processes affected by perturbations in single-cell CRISPR screening

In the format provided by the authors and unedited

Supplementary Information

Table of Contents

Supplementary Figures **2**

Supplementary Tables **10**

Supplementary Notes **13**

1. *Model specification and inference* *13*
2. *Input pre-processing* *15*
3. *Alternative GSFA models* *16*
4. *Selecting the number of factors in GSFA* *17*
5. *GSFA implementation and running time* *17*
6. *Simulation study* *17*

Supplementary Figures

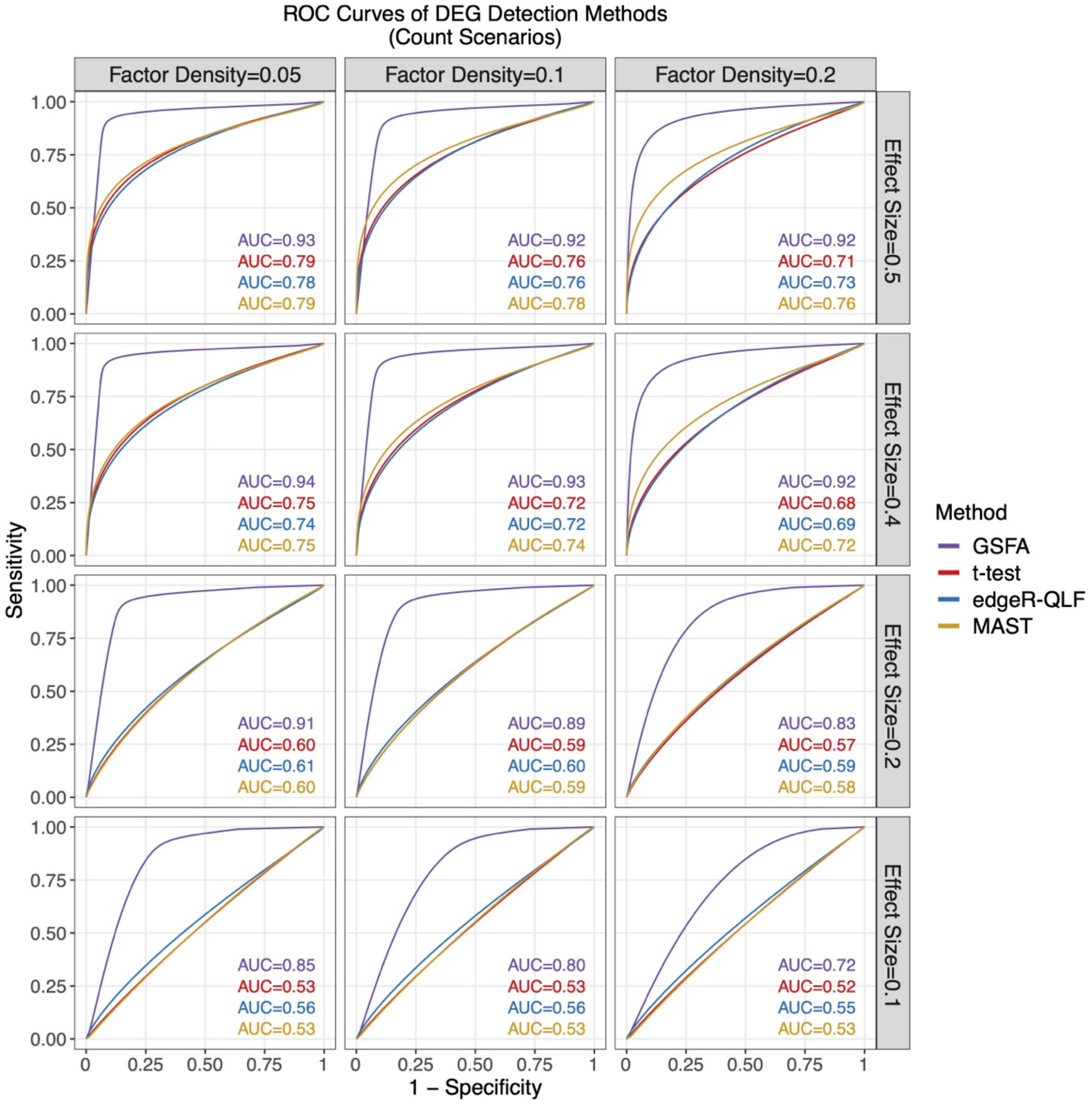

Supplementary Fig. S1: ROC curves of DEG discovery across methods on count-based simulated data. Results are across 3 different levels of true factor density, and 4 different values of true perturbation effects; 4 colors correspond to 4 DEG detection methods.

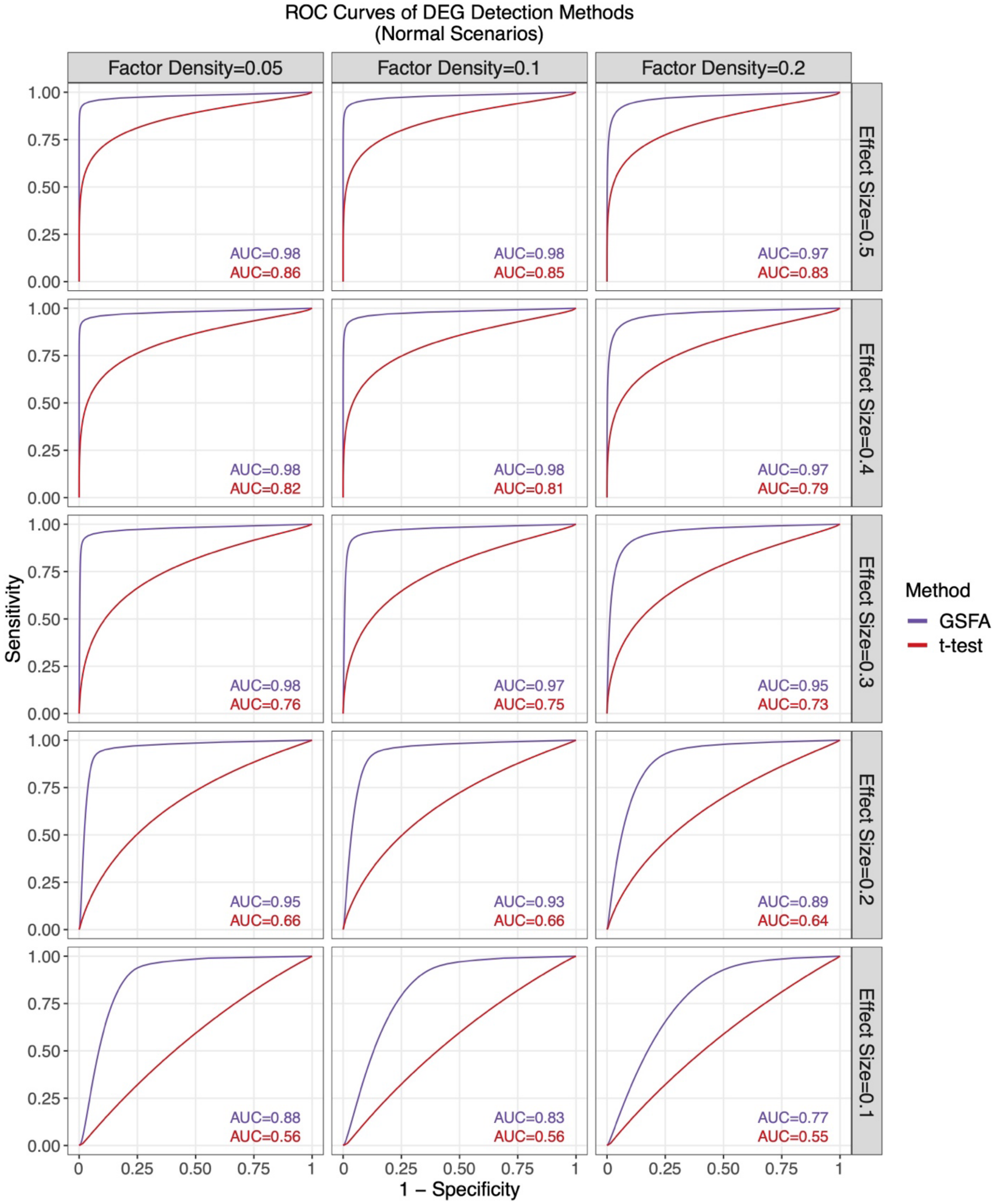

Supplementary Fig. S2: ROC curves of DEG discovery across methods on normal scenario simulated data. Results are across 3 different levels of true factor density, and 5 different values of true perturbation effects; 2 colors correspond to 2 DEG detection methods.

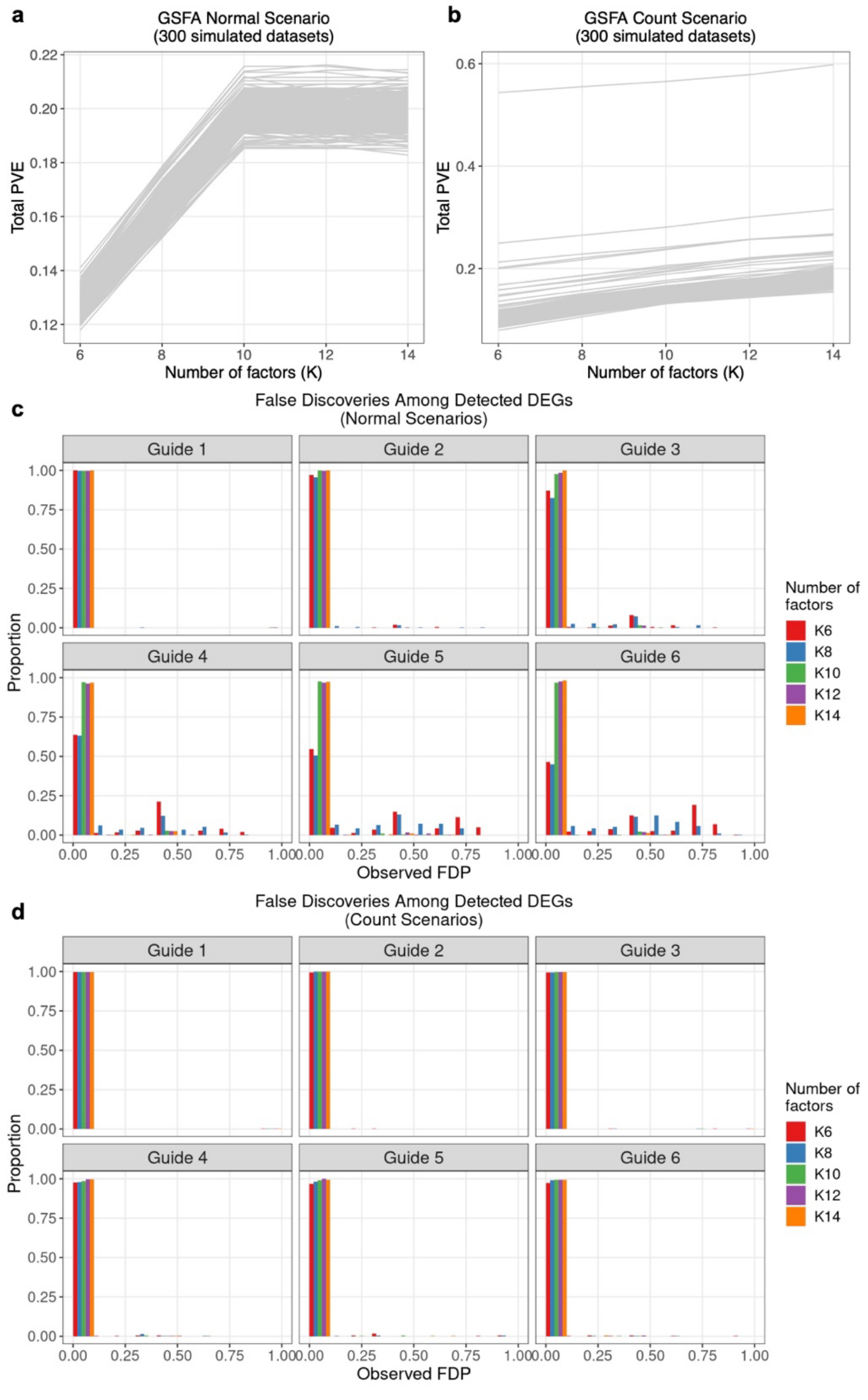

Supplementary Fig. S3: Selection of the number of factors (K) in GSFA simulations. **a)**, **b)** The percent of variation of gene expression explained (PVE) by the factors, as a function of the number of factors. Each line represents the result of one simulation out of 300. **a)** Normal simulation. **b)** Count-based simulation. **c)**, **d)** Calibration of LFSR in simulations. Each panel shows DE analysis of one of six perturbations. In each simulation, the proportion of false DE genes was obtained, and the plot shows the histogram of the proportions across 300 simulations. Each color represents a value of K. **c)** Normal simulation. **d)** Count-based simulation.

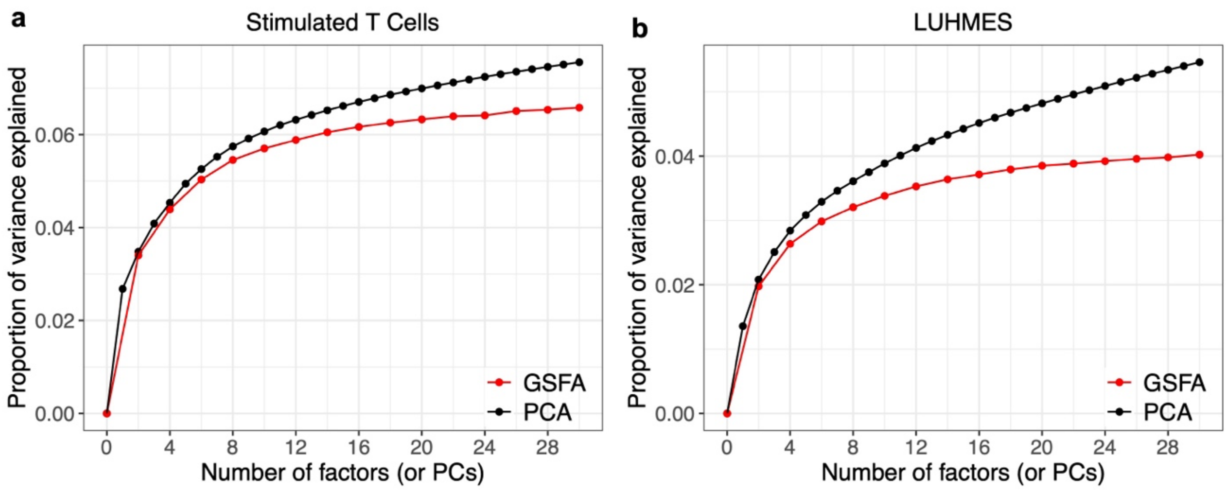

Supplementary Fig. S4: Percentage of variance explained (PVE) as a function of the number of factors or PCs for GSFA and standard PCA. **a)** T cells. **b)** LUHMES.

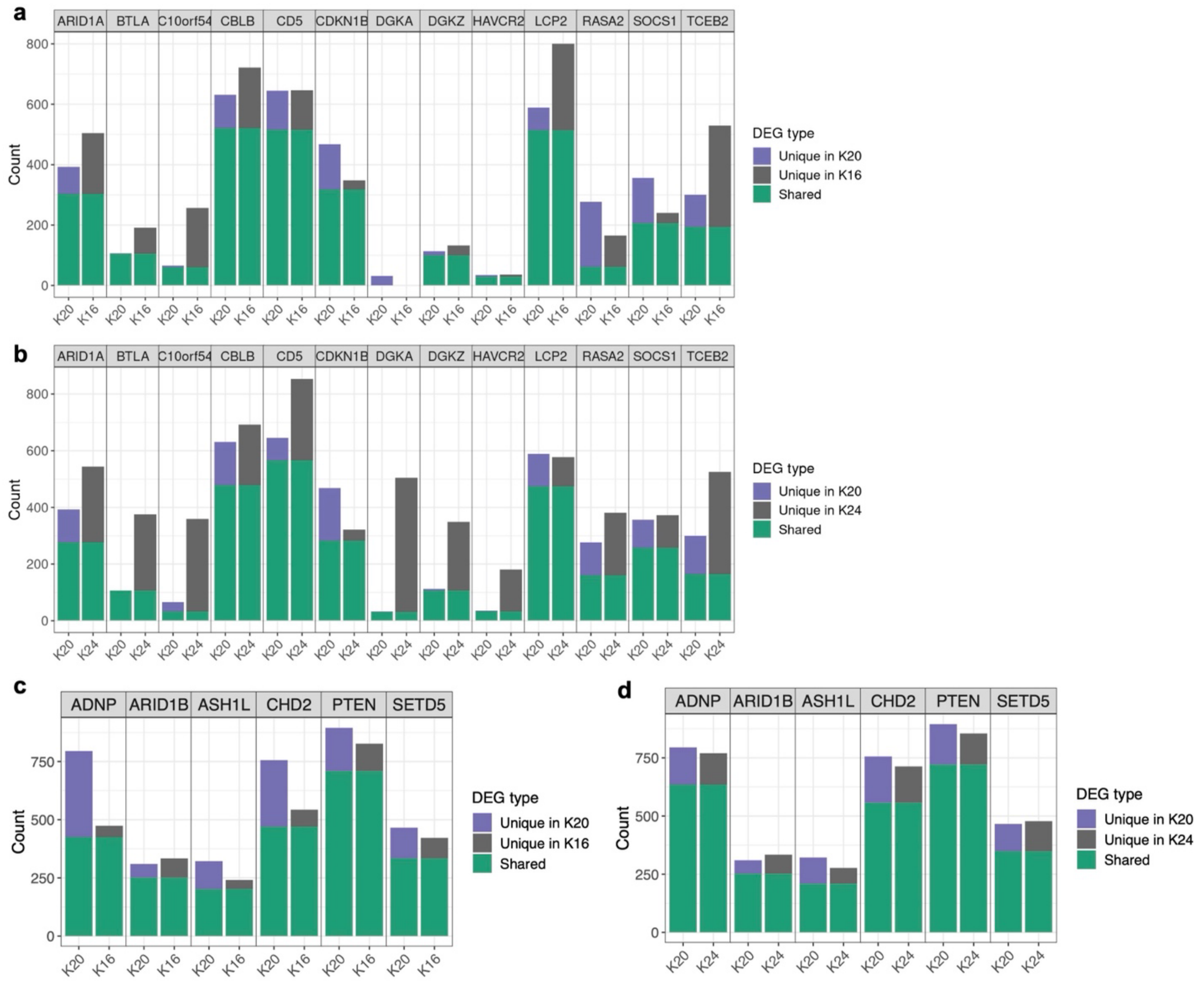

Supplementary Fig. S5: DEG results at different values of *K*, the number of factors. **a)** T cell results: *K* = 20 vs. *K* = 16. **b)** T cell results: *K* = 20 vs. *K* = 24. **c)** LUHMES results: *K* = 20 vs. *K* = 16. **d)** LUHMES results: *K* = 20 vs. *K* = 24. Only perturbed genes with a significant number (> 30) of DEGs are shown.

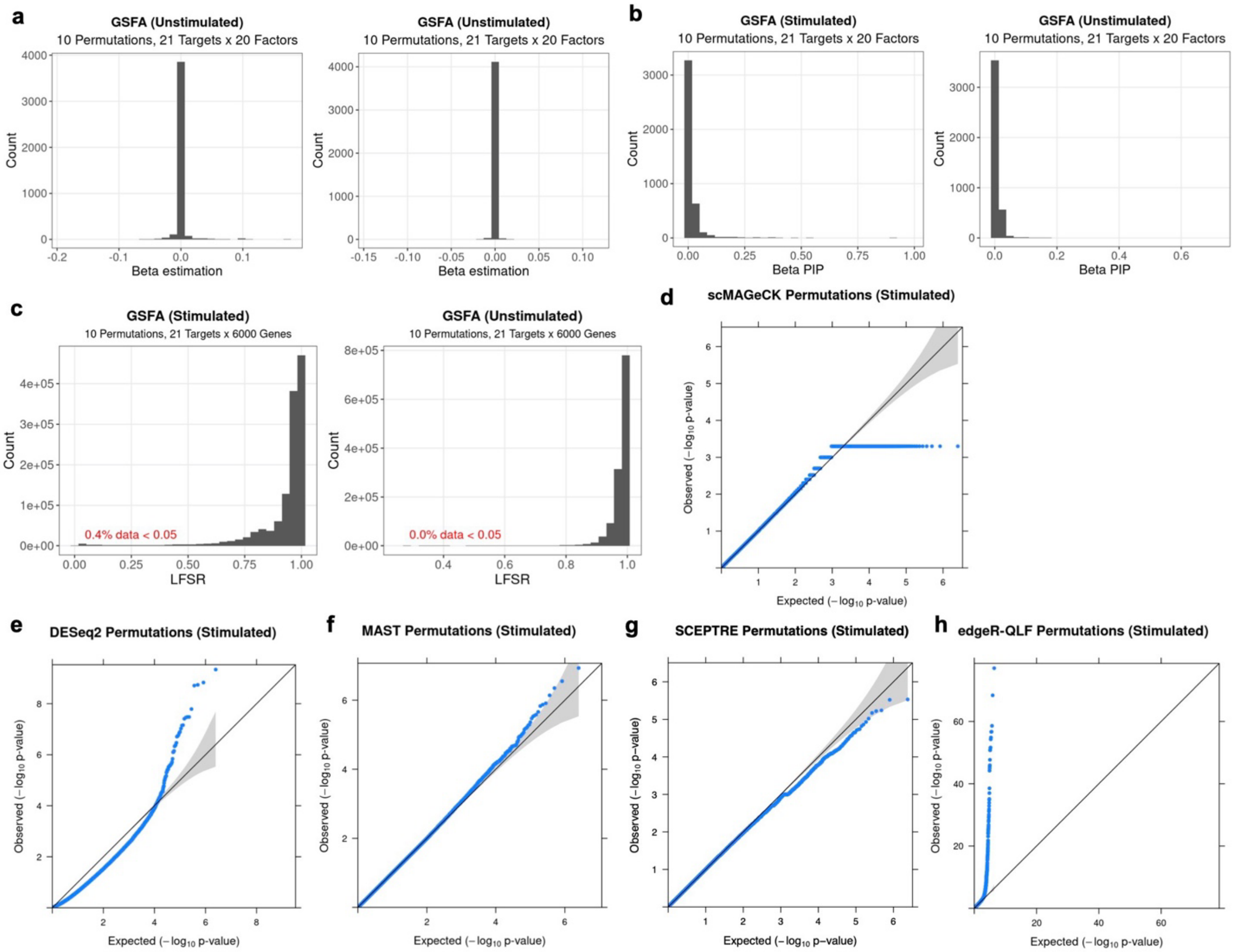

Supplementary Fig. S6: Permutation results of DEG detection methods on CD8^{+} T cell CROP-seq data. Results from 10 randomly permuted datasets are presented together. **a)** GSFA effect sizes of perturbations on factors, estimated within stimulated cells and unstimulated cells, respectively. **b)** GSFA PIPs of associations between factors and perturbations, estimated within stimulated cells and unstimulated cells, respectively. **c)** GSFA LFSRs of genes under all perturbations, estimated within stimulated cells and unstimulated cells. **d)** Quantile-quantile plot of empirical *p*-values of differential expression estimated by scMAGeCK-LR within stimulated cells, assuming a uniform(0,1) null distribution; empirical *p*-values of exact zeros were replaced with 5e-4 for visualization in the Q-Q plot (specific to (d)); the shaded band is formed by upper- and lower-bounds of the 95% confidence interval at each of the 1000-quantiles of the uniform(0,1) distribution. **e)**, **f)**, **g)**, **h)** Same as in (d) except that the observed quantile values are of differential expression *p*-values estimated within stimulated cells by DESeq2 (e), MAST (f), SCEPTRE (g), and edgeR-QLF test (h).

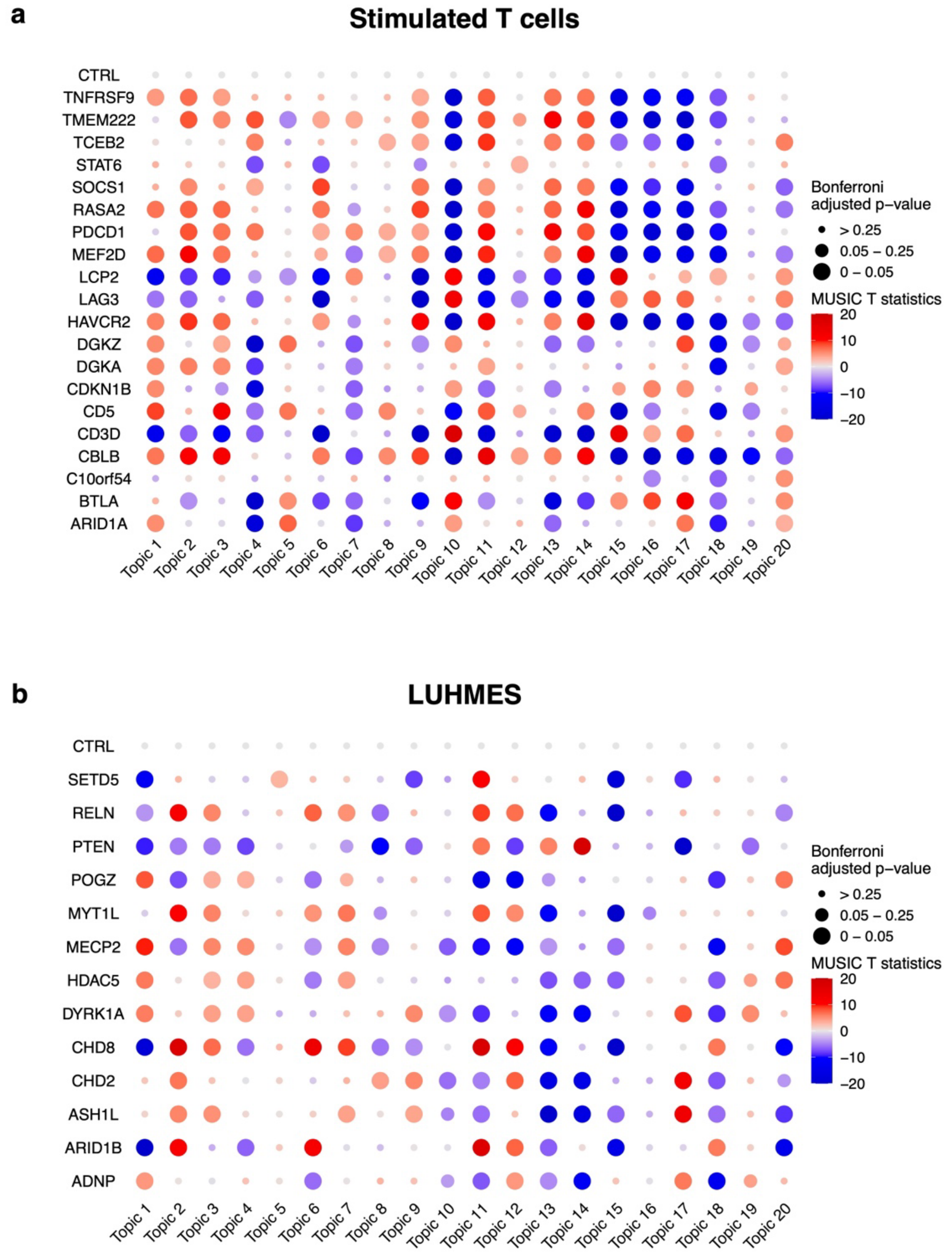

Supplementary Fig. S7: MUSIC results in real data. The heat-map shows the strength of association between a perturbed gene (row) and a topic (column). To obtain the perturbation effects on inferred topics, we adapted the MUSIC's Diff_topic_distri() function to obtain the *t*-test statistics. To evaluate the significance of the observed effects, we performed 10,000 permutations of the perturbation conditions, and computed two-sided empirical *p*-values based on how extreme the *t* statistics calculated in actual data are relative to the empirical *t* statistics distributions. Bonferroni corrections of the empirical *p*-values were made to adjust for multiple comparisons. **a)** T cell results. **b)** LUHMES results.

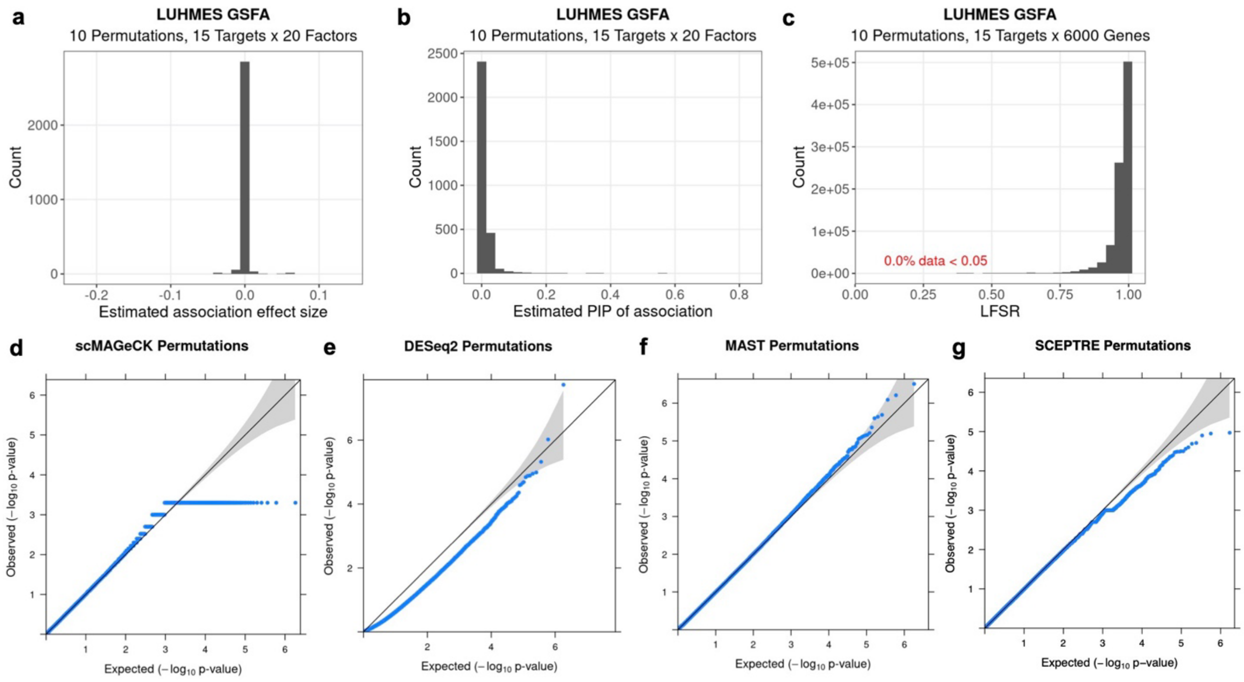

Supplementary Fig. S8: Permutation results of DEG detection methods on LUHMES CROP-seq dataset. Results from 10 randomly permuted datasets are presented together. **a)** GSFA effect sizes of perturbations on factors. **b)** GSFA estimated PIPs of associations between factors and perturbations. **c)** GSFA estimated LFSRs of genes under all perturbations. **d)** Quantile-quantile plot of empirical *p*-values of differential expression estimated by scMAGeCK-LR, assuming a uniform(0,1) null distribution; empirical *p*-values of exact zeros were replaced with 5e-4 for visualization in the Q-Q plot (specific to (d)); the shaded band is formed by upper- and lower-bounds of the 95% confidence interval at each of the 1000-quantiles of the uniform(0,1) distribution. **e)**, **f)**, **g)** Differential expression *p*-values estimated by DESeq2 (e), MAST (f), and SCEPTRE (g).

Supplementary Tables

Table S2. Full gene ontology enrichment results in T cell GSFA factors and **Table S4. Full gene ontology enrichment results in LUHMES GSFA factors** can be found in separate extended files.

Table S1. T cell marker genes

**Gene**	**Protein (Alias)**	**Annotation**	**References**
IL7R	Interleukin-7 receptor (CD127)	T cell resting state	PMID: 15308108
CCR7	CC chemokine receptor 7	T cell resting state	PMID: 11145663
GZMB	Granzyme B	T cell activation	PMID: 12360212, PMID: 22084442
IFNG	Interferon gamma	T cell activation	PMID: 11145690
CD44		T cell activation	PMID: 12526810
IL2RA	Interleukin-2 receptor	T cell activation	PMID: 18417224
XCL1	X-C motif chemokine ligand 1	T cell activation	PMID: 19913446
TNFRSF18	Glucocorticoid-induced TNFR-related protein (GITR)	T cell activation	PMID: 21076066
ITGAL	Integrin subunit alpha L (LFA-1)	T cell activation	PMID: 29774029
MKI67	Marker of proliferation Ki-67	Cell proliferation	PMID: 29322240
CENPF	Centromere protein F	Cell proliferation	PMID: 16565862
TOPBP1	DNA topoisomerase II binding protein 1	Cell proliferation	PMID: 15195143

Table S3. Neuronal marker genes

Gene	Protein (Alias)	Annotation	References
TP53	Tumor protein p53	Cell proliferation	PMID: 18948956
CDK4	Cyclin dependent kinase 4	Cell proliferation	PMID: 19733543
NES	Nestin	Neural progenitor cell	PMID: 29541793
STMN2	Stathmin-2	Mature neuron	PMID: 14598370
MAP2	Microtubule associated protein 2	Mature neuron	PMID: 10704996
DPYSL3	Dihydropyrimidinase like 3	Mature neuron	GO:0010976
MAP1B	Microtubule associated protein 1B	Mature neuron	GO:0010976
CRABP2	Cellular retinoic acid binding protein 2	Mature neuron	GO:0010976
NEFL	Neurofilament Light Chain	Mature neuron	GO:0010976
ZEB2	Zinc finger E-box binding homeobox 2	Mature neuron	GO:0010976
ITM2C	Integral membrane protein 2C	Negative regulation of neuron projection	GO:0010977
CNTN2	Contactin-2	Negative regulation of neuron projection	GO:0010975
DRAXIN	Dorsal inhibitory axon guidance protein	Negative regulation of neuron projection	GO:0010977, PMID: 24832731
HDAC2	Histone deacetylase 2	Negative regulation of neuron projection	GO:0010977
GO:0010975 regulation of neuron projection development			
GO:0010976 positive regulation of neuron projection development			
GO:0010977 negative regulation of neuron projection development			

Table S5. Running time and memory requirements of single-cell CRISPR screen methods when applied to the LUHMES dataset.

	GSFA	SCEPTRE	MUSIC
**Run time**	0.5 hr (preprocessing)<br>+<br>2.5 hrs (for 20 factors)	1 min (preprocessing)<br>+<br>2.2 hrs	1.6 hr (preprocessing)<br>+<br>2.5 hrs (for 20 topics)
**# of CPUs used**	1	10	10
**Memory**	50 Gb	50 Gb	50 Gb

Table S6. Effect size matrix (β) in a simulation scenario that include negative control cells as an additional perturbation group.

	Factor 1	Factor 2	Factor 3	Factor 4	Factor 5	Factor 6	Factor 7	Factor 8	Factor 9	Factor 10
**Guide 1**	0.1	0	0	0	0.1	0	0	0	0	0
**Guide 2**	0	0.2	0	0	0.1	0	0	0	0	0
**Guide 3**	0	0	0.3	0	0.1	0	0	0	0	0
**Guide 4**	0	0	0	0.4	0.1	0	0	0	0	0
**Guide 5**	0	0	0	0	0.5+0.1	0	0	0	0	0
**Guide 6**	0	0	0	0	0.1	0.6	0	0	0	0
**Guide 7 (NegCtrl)**	0	0	0	0	0.1	0	0	0	0	0

Table S7. Effect size matrix (β) in simulation scenarios where each of the six perturbations affects three out of ten factors.

	Factor 1	Factor 2	Factor 3	Factor 4	Factor 5	Factor 6	Factor 7	Factor 8	Factor 9	Factor 10
**Guide 1**	0.4	0.4	0.4	0	0	0	0	0	0	0
**Guide 2**	0	0.4	0.4	0.4	0	0	0	0	0	0
**Guide 3**	0	0	0.4	0.4	0.4	0	0	0	0	0
**Guide 4**	0	0	0	0.4	0.4	0.4	0	0	0	0
**Guide 5**	0.4	0	0	0	0.4	0.4	0	0	0	0
**Guide 6**	0.4	0.4	0	0	0	0.4	0	0	0	0

Supplementary Notes

1 Model specification and inference

1.1 Additional prior specification in GSFA

In line with the Bayesian framework, we specify the following conjugate prior distributions for parameters $\boldsymbol{\psi}$, $\boldsymbol{\pi}$, $\boldsymbol{\sigma}^2$, $\boldsymbol{c}^2$, $\boldsymbol{p}$, and $\boldsymbol{d}^2$ in the GSFA model:

$$\psi_j^{-1} \sim \text{Gamma}(g_0, h_0) \tag{1}$$
$$\pi_k \sim \text{Beta}(s_w r_w, s_w(1-r_w)) \tag{2}$$
$$\sigma_k^{-2} \sim \text{Gamma}(g_w, h_w) \tag{3}$$
$$c_k^{-2} \sim \text{Gamma}(g_c, h_c) \tag{4}$$
$$p_m \sim \text{Beta}(s_b r_b, s_b(1-r_b)) \tag{5}$$
$$d_m^{-2} \sim \text{Gamma}(g_b, h_b) \tag{6}$$

In practice, these hyperparameters are set to the following values: $g_0 = 1, h_0 = 1, s_w = 50, r_w = 0.2, g_w = 1, h_w = 1, g_c = 3, h_c = 0.5, r_b = 0.2, g_b = 1, h_b = 1$. In the simulation study, $s_b = 5$; in real data applications, $s_b = 20$.

By choosing these hyperparameters, we set the mean values of the prior distributions of our parameters $\bar{\pi}_k = 0.2$, $\bar{p}_m = 0.2$, $\bar{\psi}_j = \bar{\sigma}_k^2 = \bar{d}_m^2 = 1$, and $\bar{c}_k^2 = 1/6$.

1.2 Gibbs sampling steps in GSFA

Here we describe the details of the Gibbs sampling steps in GSFA.

To obtain posterior samples for β_{mk} and γ_{mk}, we first sample γ_{mk} based on the product of two ratios: the ratio of two marginal likelihoods, and the prior ratio.

$$\begin{aligned}\frac{P(\gamma_{mk}=1|\cdot)}{P(\gamma_{mk}=0|\cdot)} &= \frac{P(\mathbf{Z}|\gamma_{mk}=1, \mathbf{G}, \boldsymbol{\beta}_{-mk}, \boldsymbol{\gamma}_{-mk}, \boldsymbol{d}^2)}{P(\mathbf{Z}|\gamma_{mk}=0, \mathbf{G}, \boldsymbol{\beta}_{-mk}, \boldsymbol{\gamma}_{-mk}, \boldsymbol{d}^2)} \cdot \frac{P(\gamma_{mk}=1|p_m)}{P(\gamma_{mk}=0|p_m)} \\ &= \sqrt{\frac{L_{mk}}{d_m^2}} \exp\Big(\frac{\mu_{mk}^2}{2L_{mk}}\Big) \cdot \frac{p_m}{1-p_m},\end{aligned} \tag{7}$$

where $\mu_{mk} = L_{mk} \sum_{i=1}^N G_{im}(Z_{ik} - \sum_{l:l\neq m} G_{il}\beta_{lk})$ and $L_{mk} = (\sum_{i=1}^N G_{im}^2 + \frac{1}{d_m^2})^{-1}$.

With γ_{mk} sampled, we can obtain posterior samples of β_{mk} with

$$\beta_{mk}|\gamma_{mk} = 1 \sim N(\mu_{mk}, L_{mk}), \tag{8}$$

$$\beta_{mk}|\gamma_{mk} = 0 \sim \delta_0. \tag{9}$$

For the remaining parameters, we can obtain their posterior samples as follows:

$$W_{j\cdot}|\cdot \sim N((\mathbf{Z}^T\mathbf{Z} + \mathbf{D}_j)^{-1}\mathbf{Z}^T Y_{\cdot j}, \psi_j(\mathbf{Z}^T\mathbf{Z} + \mathbf{D}_j)^{-1}), \tag{10}$$

$$\text{where } \mathbf{D}_j = \text{diag}\Big(\frac{\psi_j}{\sigma_1^2[F_{j1} + (1 - F_{j1})c_1^2]}, ..., \frac{\psi_j}{\sigma_K^2[F_{jK} + (1 - F_{jK})c_K^2]}\Big),$$

$$F_{jk}|\cdot \sim \text{Bern}\Big(\frac{r_{jk}}{r_{jk} + 1}\Big), \text{ where } r_{jk} = \frac{\pi_k}{1 - \pi_k} c_k \exp\Big[\frac{W_{jk}^2}{2\sigma_k^2}(\frac{1}{c_k^2} - 1)\Big], \tag{11}$$

$$Z_{i\cdot}|\cdot \sim N(\mu_i, \Sigma), \tag{12}$$

$$\text{where } \mu_i = \Sigma \cdot (\mathbf{W}^T\mathbf{\Psi}^{-1}Y_{i\cdot} + \boldsymbol{\beta}G_i), \text{ and } \Sigma = (\mathbf{W}^T\mathbf{\Psi}^{-1}\mathbf{W} + \mathbf{I}_K)^{-1},$$

$$\psi_j|\cdot \sim \text{InverseGamma}\Big(g_0 + \frac{N}{2}, h_0 + \frac{1}{2}\sum_{i=1}^{N}(Y_{ij} - \sum_{k=1}^{K} Z_{ik}W_{jk})^2\Big) \tag{13}$$

$$\pi_k|\cdot \sim \text{Beta}(s_w r_w + \sum_{j=1}^{P} F_{jk}, s_w(1 - r_w) + P - \sum_{j=1}^{P} F_{jk}) \tag{14}$$

$$\sigma_k^2|\cdot \sim \text{InverseGamma}\Big(g_w + \frac{P}{2}, h_w + \frac{1}{2}\sum_{j=1}^{P}\frac{W_{jk}^2}{F_{jk} + (1 - F_{jk})c_k^2}\Big) \tag{15}$$

$$c_k^2|\cdot \sim \text{InverseGamma}\Big(g_c + \frac{1}{2}\sum_{j=1}^{P}(1 - F_{jk}), h_c + \frac{1}{2}\sum_{j:F_{jk}=0}\frac{W_{jk}^2}{\sigma_k^2}\Big) \tag{16}$$

$$p_m|\cdot \sim \text{Beta}(s_b r_b + \sum_{k=1}^{K}\gamma_{mk}, s_b(1 - r_b) + K - \sum_{k=1}^{K}\gamma_{mk}) \tag{17}$$

$$d_m^2|\cdot \sim \text{InverseGamma}\Big(g_b + \frac{1}{2}\sum_{k=1}^{K}\gamma_{mk}, h_b + \frac{1}{2}\sum_{1=k}^{K}\beta_{mk}^2\Big) \tag{18}$$

Below is the pseudocode of a basic Gibbs sampling procedure in GSFA.

Algorithm 1 Basic GSFA Gibbs sampling

Input: $\mathbf{Y}$, $\mathbf{G}$, K (the number of factors), T (the number of iterations)

Output: posterior samples and means of parameters $\{\boldsymbol{\beta}, \boldsymbol{\gamma}, \mathbf{Z}, \mathbf{W}, \ldots, \boldsymbol{c}\}$, LFSR

Initialize parameters $\boldsymbol{\beta}^{(0)}$, $\boldsymbol{\gamma}^{(0)}$, $\mathbf{Z}^{(0)}$, $\mathbf{W}^{(0)}$, $\ldots$, $\boldsymbol{c}^{(0)}$

for $t \leftarrow 1$ to T **do**

  $\boldsymbol{\beta}^{(t)}$ and $\boldsymbol{\gamma}^{(t)} \sim p(\boldsymbol{\beta}, \boldsymbol{\gamma}|\mathbf{Z}^{(t-1)}, \mathbf{W}^{(t-1)}, \ldots, \boldsymbol{c}^{(t-1)})$

  $\mathbf{Z}^{(t)} \sim p(\mathbf{Z}|\boldsymbol{\beta}^{(t)}, \boldsymbol{\gamma}^{(t)}, \mathbf{W}^{(t-1)}, \ldots, \boldsymbol{c}^{(t-1)})$

  $\ldots$

  $\boldsymbol{c}^{(t)} \sim p(\boldsymbol{c}|\boldsymbol{\beta}^{(t)}, \boldsymbol{\gamma}^{(t)}, \mathbf{Z}^{(t)}, \mathbf{W}^{(t)}, \ldots)$

end for

Estimate posterior means of parameters $\{\boldsymbol{\beta}, \boldsymbol{\gamma}, \mathbf{Z}, \mathbf{W}, \ldots, \boldsymbol{c}\}$

Compute LFSR based on posterior samples of $\boldsymbol{\beta}$, $\mathbf{W}$, $\mathbf{F}$

return posterior samples and means of all parameters, LFSR

In practice, $\mathbf{Z}$ and $\mathbf{W}$ are initialized from a truncated singular value decomposition (SVD) of the normalized gene expression matrix $\mathbf{Y}$, with the number of left (and right) singular vectors being K, the number of factors specified in the model. The last 20% quantile of elements (in terms of absolute value) in $\mathbf{W}$ are set to 0, and $\mathbf{F}$ is initialized as the binarized version of $\mathbf{W}$. β_m is initialized as the coefficients of linear regression $\mathbf{Z} \sim G_m$ $(1 \leq m \leq M)$. The last 50% quantile of elements (in terms of absolute value) in $\boldsymbol{\beta}$ are set to 0, and $\boldsymbol{\gamma}$ is initialized as the binarized version of $\boldsymbol{\beta}$.

The initialization of additional parameters is as follows: $\psi_j^{(0)} = 1$, $\pi_k^{(0)} = 0.2$, $\sigma_k^{2(0)} = 1$, $c_k^{2(0)} = 0.25$, $p_m^{(0)} = 0.2$, $d_m^{2(0)} = 1$ $(1 \leq j \leq P,\ 1 \leq k \leq K,\ 1 \leq m \leq M)$.

2 Input pre-processing

2.1 Deviance residual transformation and feature selection for count data

To accommodate the application of GSFA on count data, we follow the transformation proposed in [1], where the count data are transformed into continuous quantities in the form of deviance residuals. In a standard data normalization pipeline, raw counts are normalized by sample-specific size factors, and then log-transformed. However, due to the large number of zeros in scRNA-seq UMI counts, normalization schemes commonly used for bulk RNA-seq data may result in unstable normalization [2], and the arbitrary pseudocount added during the log transformation of exact zeros may introduce systematic errors and cause spurious differences in expression [3]. The deviance residual transformation circumvents these difficulties by directly modeling the raw count data under a multinomial null model of constant gene expression across all cells, and quantifying the fit of data in the form of deviance residuals, a quantity analogous to z-scores and approximately follow a normal distribution. Specifically, the deviance residual for gene j in cell i is

$$r_{ij} = \text{sign}(c_{ij} - \hat{\mu}_{ij})\sqrt{2c_{ij}\log\frac{c_{ij}}{\hat{\mu}_{ij}} + 2(n_i - c_{ij})\log\frac{n_i - c_{ij}}{n_i - \hat{\mu}_{ij}}}. \tag{19}$$

Here c_{ij} is the raw gene count, n_i is the library size of cell i, and $\hat{\mu}_{ij} = n_i \frac{\sum_i c_{ij}}{\sum_i n_i}$ is the expression of gene j under the null model of constant expression.
Following [1], we use an approximate multinomial deviance statistic to evaluate the deviance of a gene from the null model:

$$D_j = \sum_{i=1}^{N} r_{ij}^2. \tag{20}$$

Genes with constant expression across cells are not informative and will have a deviance of 0, while genes that vary across cells in expression will have a larger deviance. Therefore, one can pick the genes with high deviance during feature selection as an alternative to selecting highly variable genes, with the advantage that the selection is not sensitive to normalization.

3 Alternative GSFA models

3.1 GSFA model with alternative prior on gene weights

GSFA also allows one to use the standard spike-and-slab prior for the gene weights, although we find that it does not work as well as the default mixture-of-normal prior (Equation (4) in Methods). The alternative spike-and-slab prior is given by:

$$W_{jk} \sim \pi_k N(0, \sigma_k^2) + (1 - \pi_k)\delta_0. \tag{21}$$

Similarly, we introduced a latent binary matrix $\mathbf{F}_{P\times K}$ to indicate whether W_{jk}'s are nonzero. We can obtain the posterior samples of these parameters as follows:

$$\frac{P(F_{jk} = 1|\cdot)}{P(F_{jk} = 0|\cdot)} = \sqrt{\frac{\lambda_{jk}}{\sigma_k^2}} \exp\Big(\frac{\nu_{jk}^2}{2\lambda_{jk}}\Big) \cdot \frac{\pi_k}{1 - \pi_k}, \tag{22}$$

where $\nu_{jk} = \lambda_{jk} \sum_{i=1}^N Z_{ik}(Y_{ij} - \sum_{h:h\neq k} Z_{ih} W_{jh})/\psi_j$ and $\lambda_{jk} = (\sum_{i=1}^N Z_{ik}^2/\psi_j + 1/\sigma_k^2)^{-1}$.

With F_{jk} sampled, we can obtain posterior samples of W_{jk} with

$$W_{jk}|F_{jk} = 1 \sim N(\nu_{jk}, \lambda_{jk}), \tag{23}$$
$$W_{jk}|F_{jk} = 0 \sim \delta_0. \tag{24}$$

3.2 GSFA model with multiple cell groups

In the cases when one is interested in learning about the effects of perturbations under different cell types or experimental conditions, we extend the current model (Equations (1) - (4) in Methods) so that the factors are inferred using all cells but the associations between factors and perturbations are estimated separately for each cell group. For example, assuming 2 groups of cells, group 0 and group 1, we have the conditional probability of factor matrix $\mathbf{Z}$

$$P(\mathbf{Z}|\mathbf{G}, \boldsymbol{\beta_0}, \boldsymbol{\beta_1}) = \prod_{i0\in \text{group } 0} N(Z_{i0\cdot}; \boldsymbol{\beta_0} G_{i0\cdot}, \mathbf{I}_K) \prod_{i1\in \text{group } 1} N(Z_{i1\cdot}; \boldsymbol{\beta_1} G_{i1\cdot}, \mathbf{I}_K), \tag{25}$$

where $\boldsymbol{\beta_0}$ and $\boldsymbol{\beta_1}$ are $M \times K$ matrices holding the effect sizes of perturbations on factors within group 0 cells and group 1 cells, respectively.
Each effect size matrix is still subjected to the same "spike-and-slab" prior:

$$\beta_{0mk} \sim p_{0m} N(0, d_{0m}^2) + (1 - p_{0m})\delta_0, \tag{26}$$

$$\beta_{1mk} \sim p_{1m} N(0, d_{1m}^2) + (1 - p_{1m})\delta_0. \tag{27}$$

The distributions of other model parameters remain the same.

Once we have the posterior samples of parameters and latent variables, we can similarly obtain the posterior samples of the total effects of perturbations on individual genes, θ_{mj}'s, within each cell group using Equation (5) in Methods and the corresponding β_{mk} for that cell group.

4 Selecting the number of factors in GSFA

We provide some guidance for the selection of total factor number, K, in the GSFA model. We follow a strategy that is widely used in principal component analysis (PCA). Specifically, given a model with a set number of factors, we assess the percentage of variation explained (PVE) by all the inferred factors out of total gene expression variation as:

$$\text{PVE} = \frac{\sum_{j=1}^{P} \text{Var}(ZW_{j\cdot}^T)}{\sum_{j=1}^{P} \text{Var}(Y_{\cdot j})}, \tag{28}$$

where $\text{Var}(Y_{\cdot j})$ denotes the sample variance of gene j. Y is the input cell by gene matrix for the model, which contains continuous gene expression levels in normal scenarios, or transformed deviance residuals in count-based and real data scenarios. Since we do not know the true values of Z and W, in practice, we use their posterior estimates as an approximation.

We varied the number of factors in the model, K, from 6 to 14 in simulations, and computed the PVE under each model. In all cases, we observed that PVE initially increases with K, but saturates around the true number of underlying factors, 10. We also varied K from 2 to 30 in real data applications, and observed saturation of PVE around $K = 20$, which justified our choice of 20 factors in the model. Overall, we have provided a PVE-based procedure that can help select a reasonable number of factors in GSFA application.

5 GSFA implementation and running time

The computational complexity of the GSFA inference is $O(NK + PK)$ per iteration, with N being the number of cells, K being the number of factors, and P being the number of genes. The average run time of GSFA on a simulated dataset of 4000 cells, 6000 genes, and 10 factors is 1.32 seconds per iteration on a modern Linux workstation with Intel Xeon E5-2680 v4 (2.40 GHz) processors. The running time and memory requirement of GSFA in one real dataset (LUHMES) are shown in Table S5. We also included in the table two related single-cell CRISPR analysis methods, SCEPTRE and MUSIC, for comparison.

GSFA was implemented in `R` and `Rcpp` (using the R packages `Rcpp_1.0` and `RcppArmadillo_0.10`, and is available at Github: `https://github.com/xinhe-lab/GSFA`. R packages `ggplot2_3.3.3` and `ComplexHeatmap_2.6.2` were used for visualization. All analyses in this study were performed in `R version 4.2.0`.

6 Simulation study

6.1 Simulation study using real scRNA-seq

We created an additional simulation scenario to compare GSFA and other methods for detecting target genes of perturbations. Our motivations are two fold. First, we would like to use a real

scRNA-seq dataset to test GSFA. Secondly, we would like to test how robust GSFA is when its data generative model is not followed. So we will avoid using latent factors in our simulation procedure. We downloaded a real scRNA-seq dataset of Peripheral Blood Mononuclear Cells (PBMCs) from 10X Genomics: `https://github.com/10XGenomics/single-cell-3prime-paper/tree/master/pbmc68k_analysis`. This dataset is widely used for benchmarking of scRNA-seq analysis methods. We focused on "CD19+ B cells", which were labeled by the original study. After performing quality control, our expression matrix contained 10,830 genes and 5,866 cells.

We introduced three true gRNAs and one negative control gRNA into the expression data. To achieve this, we randomly perturbed 10% of cells, and chose 300 target genes for each gRNA. All target genes were selected from the set of highly expressed genes, defined as genes expressed in over 10% of cells. We placed the gRNAs in these cells in a way to ensure that each cell contains at most one gRNA. Furthermore, the target gene sets of each gRNA were disjoint.

To simulate the effect of a true gRNA, we perturbed the expression of its target genes in each cell containing that gRNA. First, we log-normalized the read count matrix using the median library size of 1294 as the scale factor. We then sampled the effect sizes of the three gRNAs from Normal distributions with mean zero. The standard deviations of the effect sizes were set at 0.3, 0.4, and 0.5 for the three gRNAs, respectively. After introducing the gRNA perturbations, we transformed the normalized expression matrix back to read counts and rounded all values to the closest integer. This resulting read count matrix was used as input for three methods we compared, namely GSFA, MAST, and Wilcoxon rank sum test.

For the differential expression analysis, we compared cells perturbed by a true gRNA with those perturbed by the negative control gRNA. To select differentially expressed genes (DEGs), we used a local false sign rate (LFSR) cutoff of below 0.05 for GSFA, and a false discovery rate (FDR) cutoff of below 0.05, using the Benjamini-Hochberg method for multiple testing correction, for MAST and Wilcoxon.

6.2 Evaluation of other methods on simulated data

For comparison, we have run other differential gene expression (DGE) methods. We applied Welch's t-test [4] to both the normal data and count-normalized deviance residual data. For count data scenarios, we also applied edgeR-QLF [5] and MAST [6]. In all these differential expression tests, cells with each perturbation were compared with all other cells without this perturbation for all genes, FDR was computed following the Benjamini-Hochberg procedure for genes under each perturbation, and significant DEGs were obtained by thresholding FDR < 0.05.

We also compared GSFA with a two-step clustering procedure. In the first step, we clustered the cells based on expression of all genes, and in the next step, we detected clusters significantly associated with each of the perturbations. We assessed the results in two ways. First, we assessed the power of discovering the effects of perturbations. Note that it is not straightforward to map clusters with factors in simulation data. For simplicity, we consider the association of a perturbation with any cluster as a true positive. This metric favors clustering-based analysis. Second, to define the target DEGs of a perturbation, we first obtained genes differentially expressed between each cluster and the rest, and then took the union of all DEGs of the clusters associated with the perturbation of interest. We then compared the resulting DEGs with the true target genes of

the perturbations. This approach was applied to both normal and count-based simulations. In the normal simulation, we used the K-means algorithm to cluster, and used the t-test to identify DEGs of each cluster. In the count-based simulations, we used Seurat to cluster cells and identified DEGs of each cluster using MAST. To make the results comparable to GSFA, we set the number of clusters at 10 (i.e., the true number of factors used in the simulations).

We also evaluated a two-step factor analysis procedure, where one first performs factor analysis on expression data, then associates the perturbations with factors by linear regression. This allowed us to associate a perturbation with a set of factors, at FDR < 0.05. For factor analysis, we used an "unguided" version of GSFA, where we run GSFA with the perturbation matrix ($\mathbf{G}$) set at 0. This way, the perturbation information is not used in factor analysis, and GSFA reduces to a sparse factor analysis model. To identify target genes of a perturbation, we took the union of all genes loaded to the factors (at PIP > 0.95) associated with the perturbation.

References

1. Townes, F. W., Hicks, S. C., Aryee, M. J. & Irizarry, R. A. Feature selection and dimension reduction for single-cell RNA-Seq based on a multinomial model. *Genome Biology* **20,** 1–16. ISSN: 1474760X (Dec. 2019).
2. Lun, A. T., Bach, K. & Marioni, J. C. Pooling across cells to normalize single-cell RNA sequencing data with many zero counts. *Genome Biology* **17,** 1–14. ISSN: 1474760X (Apr. 2016).
3. Lun, A. Overcoming systematic errors caused by log-transformation of normalized single-cell RNA sequencing data. *bioRxiv,* 404962. ISSN: 2692-8205. `https://www.biorxiv.org/content/10.1101/404962v1` (Aug. 2018).
4. Welch, B. L. The generalisation of student's problems when several different population variances are involved. *Biometrika* **34,** 28–35. ISSN: 0006-3444 (1947).
5. Robinson, M. D. & Smyth, G. K. Moderated statistical tests for assessing differences in tag abundance. *Bioinformatics* **23,** 2881–2887. ISSN: 1367-4803 (Nov. 2007).
6. Finak, G. *et al.* MAST: A flexible statistical framework for assessing transcriptional changes and characterizing heterogeneity in single-cell RNA sequencing data. *Genome Biology* **16,** 1–13. ISSN: 1474760X (Dec. 2015).
