# Supplementary material for: A new Bayesian factor analysis method improves detection of genes and biological processes affected by perturbations in single-cell CRISPR screening
Source: Nat Methods. 2023 Sep 28;20(11):1693–703. doi: 10.1038/s41592-023-02017-4 (PMC10630124; doi:10.1038/s41592-023-02017-4)
Supplement: Supplementary file 2 — Reporting Summary [file 41592_2023_2017_MOESM2_ESM.pdf]

nature portfolio

Corresponding author(s): Mengjie Chen, Xin He

Last updated by author(s): Jun 25, 2023

Reporting Summary

Nature Portfolio wishes to improve the reproducibility of the work that we publish. This form provides structure for consistency and transparency in reporting. For further information on Nature Portfolio policies, see our Editorial Policies and the Editorial Policy Checklist.

Statistics

For all statistical analyses, confirm that the following items are present in the figure legend, table legend, main text, or Methods section.

n/a	Confirmed	
☐	☒	The exact sample size (*n*) for each experimental group/condition, given as a discrete number and unit of measurement
☒	☐	A statement on whether measurements were taken from distinct samples or whether the same sample was measured repeatedly
☐	☒	The statistical test(s) used AND whether they are one- or two-sided<br>*Only common tests should be described solely by name; describe more complex techniques in the Methods section.*
☒	☐	A description of all covariates tested
☐	☒	A description of any assumptions or corrections, such as tests of normality and adjustment for multiple comparisons
☐	☒	A full description of the statistical parameters including central tendency (e.g. means) or other basic estimates (e.g. regression coefficient) AND variation (e.g. standard deviation) or associated estimates of uncertainty (e.g. confidence intervals)
☐	☒	For null hypothesis testing, the test statistic (e.g. *F*, *t*, *r*) with confidence intervals, effect sizes, degrees of freedom and *P* value noted<br>*Give P values as exact values whenever suitable.*
☐	☒	For Bayesian analysis, information on the choice of priors and Markov chain Monte Carlo settings
☒	☐	For hierarchical and complex designs, identification of the appropriate level for tests and full reporting of outcomes
☐	☒	Estimates of effect sizes (e.g. Cohen's *d*, Pearson's *r*), indicating how they were calculated

Our web collection on statistics for biologists contains articles on many of the points above.

Software and code

Policy information about availability of computer code

Data collection	No software was used for data collection.
Data analysis	All analyses were done in R 4.2.0. Alternative differential gene expression analysis tools used in this study includes: Welch's t-test using the t.test() function in R base package stats, R package edgeR_3.32.1, R package DESeq2_1.30.1, R package MAST_1.16.0, R package scMAGeCK_1.2.0, R package sceptre_0.1.0, and R package MUSIC_1.0. Gene ontology enrichment analysis was done using R package WebGestaltR_0.4.4. R package Seurat_4.0.1 was used for initial merging and QC of single-cell data. Visualization tools used in this study includes: R package ggplot2_3.3.3 and R package ComplexHeatmap_2.6.2. Rcpp_1.0 and RcppArmadillo_0.10 were used to implement the GSFA software.

For manuscripts utilizing custom algorithms or software that are central to the research but not yet described in published literature, software must be made available to editors and reviewers. We strongly encourage code deposition in a community repository (e.g. GitHub). See the Nature Portfolio guidelines for submitting code & software for further information.

Data

Policy information about availability of data

All manuscripts must include a data availability statement. This statement should provide the following information, where applicable:

- Accession codes, unique identifiers, or web links for publicly available datasets
- A description of any restrictions on data availability
- For clinical datasets or third party data, please ensure that the statement adheres to our policy

Both CROP-seq datasets used in this study are publicly available and were downloaded from GEO: GSE119450 and GEO: GSE142078, respectively.

Human research participants

Policy information about studies involving human research participants and Sex and Gender in Research.

Reporting on sex and gender	NA
Population characteristics	NA
Recruitment	NA
Ethics oversight	NA

Note that full information on the approval of the study protocol must also be provided in the manuscript.

Field-specific reporting

Please select the one below that is the best fit for your research. If you are not sure, read the appropriate sections before making your selection.

☒ Life sciences ☐ Behavioural & social sciences ☐ Ecological, evolutionary & environmental sciences

For a reference copy of the document with all sections, see nature.com/documents/nr-reporting-summary-flat.pdf

Life sciences study design

All studies must disclose on these points even when the disclosure is negative.

Sample size	We used published datasets, and generally followed the reported procedures to process the data. Therefore, the sample sizes in the study are almost the same as in the original studies (main text reference 10, 39), except for the difference in data preprocessing.
Data exclusions	When applying GSFA to both CROP-seq datasets, we only included the top 6000 genes ranked in decreasing deviance statistics (see Methods for detail) and filtered the rest. The number of genes would be similar if we filter genes according to their detection rates in cells at a threshold of 10%, a common practice in single-cell RNA-seq data analysis.
Replication	We generated 300 replications of simulated datasets under each simulation setting in the study, and results of all replications are presented in the manuscript. Both real CROP-seq datasets analyzed in this study are publicly available, therefore, replication does not apply for them.
Randomization	Randomization is not relevant to our study. We used publicly available CROP-seq datasets in analysis. We played no role in the experimental design of these studies.
Blinding	Blinding is not relevant to our study. We used publicly available CROP-seq datasets in analysis. We played no role in the experimental design of these studies.

Reporting for specific materials, systems and methods

We require information from authors about some types of materials, experimental systems and methods used in many studies. Here, indicate whether each material, system or method listed is relevant to your study. If you are not sure if a list item applies to your research, read the appropriate section before selecting a response.

Materials & experimental systems

n/a	Involved in the study	
☒	☐	Antibodies
☒	☐	Eukaryotic cell lines
☒	☐	Palaeontology and archaeology
☒	☐	Animals and other organisms
☒	☐	Clinical data
☒	☐	Dual use research of concern

Methods

n/a	Involved in the study	
☒	☐	ChIP-seq
☒	☐	Flow cytometry
☒	☐	MRI-based neuroimaging
